# Identification and validation of prognostic genes associated with T-cell exhaustion and macrophage polarization in breast cancer

**DOI:** 10.3389/fendo.2025.1556496

**Published:** 2025-05-27

**Authors:** Fengqiang Cui, Changjiao Yan, Jiang Wu, Yuqing Yang, Jixin Yang, Jialing Luo, Nanlin Li

**Affiliations:** Department of Thyroid, Breast and Vascular Surgery, Xijing Hospital, The Fourth Military Medical University, Xi’an, Shaanxi, China

**Keywords:** breast cancer, T-cell exhaustion, macrophage polarization, prognostic risk model, immune infiltration analysis

## Abstract

**Background:**

The most frequent malignant tumor in women is breast cancer (BRCA). It has been discovered that T-cell exhaustion and macrophages play significant roles in BRCA. It was necessary to explore prognostic genes associated with T-cell exhaustion and macrophage polarization in BRCA.

**Methods:**

The following data were included: 35 macrophage polarization-related genes (MPRGs), 683 T-cell exhaustion-related genes (TEXRGs), GSE20685, as well as TCGA-BRCA. Initially, candidate genes were identified through crossing differentially expressed genes (DEGs) obtained by differential expression analysis, key module genes associated with MPRGs, as well as TEXRGs. Next, 101 combinations of 10 machine learning algorithms and univariate Cox analysis were utilized to screen for prognostic genes. Concurrently, a risk model was built for validation in TCGA-BRCA and GSE20685. Next, we conducted immune infiltration, immunotherapy, mutation analysis, molecular regulatory network, as well as drug sensitivity between the two risk groups. Ultimately, we did the reverse transcription-quantitative polymerase chain reaction (RT-qPCR).

**Results:**

According to random survival forest (RSF) algorithm (the best combination with the greatest C-index of 0.799), 7 prognostic genes were selected, which are PGK1, BTG2, TANK, CFB, EIF4E3, TNFRSF18, and BATF. After that, we created a risk model, and in the low-risk samples, there was a relatively high survival rate. Next, between two risk parts, the 7 differential immune cells were found. There was a significant difference in 25 immunological checkpoint (ICI) genes between the two risk parts. Next, a lncRNAs-miRNA-mRNA network with 65 nodes and 70 edges was built. Additionally, 84 medications were shown to differ significantly between the two risk groups. Finally, the expression of BTG2, TANK, and EIF4E3 was verified by RT-PCR, which was consistent with the bioinformatics analysis.

**Conclusion:**

The 7 prognostic genes (PGK1, BTG2, TANK, CFB, EIF4E3, TNFRSF18, and BATF) were screened, providing new insights into potential treatments for BRCA.

## 1 Introduction

Breast cancer has overtaken lung cancer as the most commonly diagnosed cancer in women, with an estimated 2.3 million new cases each year (1). By 2040, the BRCA burden is expected to exceed 3 million new cases and 1 million deaths annually (2). Surgery, chemotherapy and radiotherapy remain the mainstay of treatment for BRCA. Thanks to advances in diagnosis and treatment, the five-year survival rate for stage I breast cancer is 99 percent, compared to only 28 percent for stage IV. In addition, BRCA is a complex and heterogeneous disease, and the treatment outcomes for different patients often vary greatly (3). Coupled with issues of drug recurrence, and metastasis, this leads to poor patient prognosis (4, 5). Therefore, the identification of genetic biomarkers for breast cancer prognosis and the elucidation of their underlying molecular mechanisms are crucial for the development of precision therapies and the improvement of patient outcomes.

The immune system plays a dual role in breast cancer, not only suppressing tumor growth but also being exploited by the tumor to promote its progression. Effective T cell responses are a critical component of adaptive immunity, protecting against both foreign pathogens and malignant transformation. However, prolonged antigen exposure leads to T-cell dysfunction, a process known as exhaustion (6). The functional decline of T cells during chronic stimulation, commonly referred as T-cell exhaustion (TEX), is a major limitation of current immunotherapy (7). Exhausted T cells are effector lymphocytes that express multiple inhibitory receptors (PD1, LAG3, CTLA4 and TIM-3) (8). The reactivation of T cells mediates anti-tumor immunity in BRCA immunotherapy (9). In addition to T cells, macrophages also play a key role in the tumor microenvironment. Macrophages are crucial cells in the human body’s innate immunity and are involved in a variety of non-inflammatory responses. There are two main forms of macrophage polarization, M1-like and M2-like, each of which can be polarized into two types under different cell stimulation conditions. The two types of macrophages secrete different cytokines during the disease process, resulting in two opposing effects (10). VISTA affects BRCA tumor cells growth by critically regulating the macrophage polarization through the STAT pathway (11). Research has found that tumor-associated macrophages expressing the transcription factor IRF8 contribute to TEX in cancer. The advent of immune checkpoint blockade (ICB) therapy restores some function to exhausted cells to fight various cancer types. Continuing to dissect the processes that regulate TEX and macrophage polarization will help design enhanced immune-mediated therapies that can reduce adverse effects and significantly improve outcomes for BRCA patients (11, 12). The emergence of immune checkpoint blockade (ICB) therapy has revolutionized cancer treatment by restoring exhausted T cell (TEX) functionality and enhancing anti-tumor immunity. However, the therapeutic efficacy of ICB in breast cancer (BRCA) remains limited due to the complex interplay between T cell exhaustion and macrophage polarization. Therefore, in-depth investigation into the regulatory mechanisms governing TEX and macrophage polarization will not only elucidate the molecular basis of tumor immune evasion but also provide a theoretical foundation for developing novel immunotherapeutic strategies.

Based on the transcription data of public databases, this study has identified BRCA prognostic genes associated with T cell exhaustion and macrophage polarization through differential expression analysis, WGCNA, univariate Cox regression analysis and machine learning methods. The immune infiltration, enrichment pathways and molecular regulatory mechanisms of prognostic genes were explored, and mutation analysis and drug sensitivity analysis were performed. This provides a new scientific idea for the prognosis of BRCA.

## 2 Materials and methods

### 2.1 Data resource

The data, including information on mutations, RNA sequencing (RNA-Seq), microRNA (miRNA)-Seq, long non-coding RNA (lncRNA)-Seq, clinical information (age, gender, stage staging, T/N/M staging), as well as survival information, were acquired from the TCGA database. Excluded patient data were those lacking survival information. The training set of TCGA-BRCA data comprised 113 normal samples and 904 tumor samples from BRCA patients (13). The inclusion and exclusion criteria were presented in **Supplementary Table 1**. The prognostic model (14) was validated through the validation set GSE20685 (GPL570), which comprised 327 breast cancer samples, which was mined from the GEO database. Additionally, the literature provided 683 T-cell exhaustion-related genes (TEXRGs) (15) and 35 macrophage polarization-related genes (MPRGs) (16). For handling missing values, we used the na.omit() function to remove missing data and ensure data integrity, preventing missing values from interfering with subsequent analysis.

### 2.2 Immune infiltration analysis in TCGA-BRCA

In TCGA-BRCA, the infiltration levels of 22 immune cells were evaluated using CIBERSORT algorithm between BRCA and normal samples (17). The distribution of immune cells was visualized using a heatmap made through ggplot2 (v3.4.4) (18), after removing 3 immune cells with a distribution ratio of zero. Then, through the Wilcoxon test, the differences in immune cells between BRCA and normal samples were analyzed (p < 0.05). Moreover, Spearman analysis (|cor| > 0.3, p < 0.05) was employed to examine the correlation between differential immune cells (19).

### 2.3 Differential expression analysis

Between cancer and normal samples (TCGA-BRCA), DESeq2 (v 1.42.0) (20) was utilized to acquired differentially expressed genes (DEGs) (p.adj < 0.05, |log_2_fold change (FC)|>0.5). After that, DEGs were visualised through volcano maps by ggplot2, and the top 10 DEGs based on |log_2_FC| were labeled. Furthermore, DEGs were displayed on a heat map by pheatmap (v1.0.12) (21). In the differential expression analysis, DESeq2 normalized the data by estimating size factors to adjust for sequencing depth differences between samples, ensuring the gene expression data across samples is comparable. Variance Stabilizing Transformation (VST) to normalize the gene expression data for differential analysis. VST converts raw count data into data that is approximately normally distributed.

### 2.4 Screening of key module genes

In the WGCNA analysis, when filtering module genes, we used the absolute values of gene significance (GS) and module membership (MMblue). We retained genes with an absolute GS value greater than 0.6 and an absolute MM value greater than 0.4. The weighted gene co-expression network analysis (WGCNA) package (v1.71) was utilized to analyze WGCNA. Initially, differentially expressed MPRGs (DE-MPRGs) were acquired by intersecting MPRGs with DEGs. Next, according to GSVA (v 1.50.0) (22), DE-MPRGs scores were assessed through single-sample genome enrichment analysis (ssGSEA). Next, according to median scores, the samples were split into high- and low-scoring parts, also overall survival (OS) survival curves were drawn between the 2 scoring groups. To guarantee the accuracy of the study, the BRCA samples in TCGA-BRCA were then grouped and outlier samples were eliminated. The optimal soft threshold was then identified. The selection criteria were met when the mean connectivity also trend toward 0 and the R^2^ exceeded the threshold value of 0.85. Subsequently, the co-expression matrix was constructed, with a minimum of 100 genes per gene module. The Pearson function was utilized to examine the correlation between each gene module and MPRGs scores, and a correlation heatmap was created (|cor| > 0.3, p < 0.05) to identify key modules with high correlation with MPRGs scores, and their genes were combined as key module genes (23).

### 2.5 Candidate gene acquisition, protein-protein interaction network construction, as well as functional enrichment analysis

Key module genes, TEXRGs, and DEGs were intersected through the ggvenn (v 0.1.10) (24)to identify potential genes related to T-cell exhaustion and macrophage polarization in BRCA patients. Next, with the help of the search tool for recurring instances of neighboring genes (STRING) database (confidence=0.4), the PPI network between the candidate genes was built to thoroughly investigate the interactions between the relevant proteins of the candidate genes (25). Cytoscape (v 3.7.1) (26) was employed to display the PPI network. Additionally, we did the gene ontology (GO) as well as the Kyoto encyclopedia of genes and genomes (KEGG) (p< 0.05).

### 2.6 Screening of prognostic genes

Using survival (v 3.5-7) (https://CRAN.R-project.org/package=survival) and the 904 BRCA samples in TCGA-BRCA, univariate Cox regression analysis of candidate genes was conducted to screen for prognostic-related genes (hazard ratios (HR) ≠ 1, p<0.01). Following this, the PH assumption testing was evaluated for the Cox regression model (incorporating survival data and candidate genes) by using the cox.zph function. Genes that passed the PH test (p > 0.05) were identified as signature genes and plotted visually by a forest plot (27). Next, 101 combinations of 10 machine learning (stepwise Cox, elastic network [Enet], random survival forest [RSF], partial least squares regression for Cox [plsRcox], survival support vector machine [survival-SVM], CoxBoost, supervised principal components [SuperPC], LASSO, Ridge, as well as generalized boosted regression modeling [GBM]) were generated to fit predictive models for TCGA-BRCA dataset and validating them in GSE20685. The optimal machine learning model (with the highest concordance index (C-index) and better prognostic value) was selected. The genes in the optimal model were labelled as prognostic genes.

### 2.7 Risk model construction and evaluation

Based on TCGA-BRCA and the median risk score, BRCA samples were categorized into high- and low-risk groups. Then, survival analysis was undertaken. Then, we produced Kaplan-Meier (KM) survival curves. Subsequently, the accuracy of the risk model was assessed through receiver operating characteristic curves (ROC) by timeROC (v 0.4) (28). Furthermore, using the GSE20685 dataset, the risk model was validated.

### 2.8 Association between risk scores and clinical characteristics

The differences in different clinical characteristics (age, tumor stage, gender, T-stage, N-stage, as well as M-stage) were contrasted through Wilcoxon test between two risk groups (p < 0.05) (29). For processing cancer staging information, we removed samples with “Stage X” using the command clin_data <- clin_data[-which(clin_data$Stage == “Stage X”)],. “Stage X” represents an unknown stage, and these samples are considered invalid or anomalous. The outcomes were drawn by violin plots. Subsequently, KM survival curves were created for the various subtypes of the aforementioned clinical characteristics, and survival differences among subtypes were contrasted through Log-rank test.

### 2.9 Independent prognostic analysis and nomogram construction

The univariate Cox regression analysis (p < 0.05), PH assumption test (27), as well as multivariate Cox regression analysis (p < 0.05) were conducted to uncover independent prognostic factors influencing BRCA patients. Next, the resulting factors were then incorporated into construction of a prognostic nomogram. Through constructing decision curve analysis (DCA), ROC curves, as well as calibration curve, we assessed the prognosis nomogram.

### 2.10 Immune infiltration analysis in high- and low-risk groups

In the two risk groups, the proportions of each immune cell were displayed following the elimination of immune cells (distribution ratio of 0). Next, the Wilcoxon test (p< 0.05) was conducted to determine the differential immune cells.

### 2.11 Immunotherapy analysis

The expression differences of 38 conventional immunological checkpoints (ICI) (30) between two risk groups of TCGA-BRCA were evaluated to examine the link between these groups and ICI. Next, the association between differential ICI genes and prognostic genes was explored. The immunological score, stromal score, as well as estimated score were then determined by the ESTIMATE algorithm (31). Lastly, according to the TIDE algorithm, the differences in TIDE, dysfunction, CD274 as well as exclusion scores were examined between the two risk groups.

### 2.12 Mutation analysis

In order to ascertain which genes were mutated in BRCA patients, the TCGA-BRCA provided the mutation data. Next, the differences in mutation rates for the top 5 mutated genes were assessed between two risk parts. After that, the variations in tumor mutation burden (TMB) and risk ratings between two risk groups were examined. Afterwards, the patients were divided into high- and low-TMB groups according to the optimal threshold for TMB scores, and the KM survival curves for each group were created. The groups classified as low risk-high TMB group, high risk-high TMB group, low risk-high TMB group, as well as high risk-low TMB group. Eventually, the Kruskal-Wallis test was utilized to examine the OS differences among the 4 groups.

### 2.13 Analysis of copy number variants

Chromosomal analysis of somatic CNV (SCNA) was carried out through the GISTIC algorithm in TCGA-BRCA. Copy number deletions and amplifications were independently quantified between the two risk groups, and the copy number differences were assessed. Subsequently, the GISTIC algorithm was employed to count the CNVs of the prognostic genes of TCGA-BRCA in BRCA patients. Furthermore, prognostic gene mutations in BRCA patients were analyzed by the cBioPortal (https://www.cbioportal.org) website.

### 2.14 Enrichment analysis of prognostic genes

Differential expression analyses were undertaken through the DESeq2 package to acquire DEGs2 between two risk groups, ranked by size of log_2_FC values. The ranked genes in the background gene set (c2.cp.kegg.v7.5.1.entrez.gmt) were analyzed through clusterProfiler for GSEA (p < 0.05). Then, the results were visualized by drawing ridge plots with the help of the GseaVis (v 0.0.5) (https://CRAN.R-project.org/package=GseaVis).

### 2.15 Regulatory network analysis

DE-lncRNA and DE-miRNAs were subjected to differential expression analysis through DESeq2 and limma (v 3.58.1) between BRCA and normal samples (32), respectively. The criteria for screening were p.adj < 0.05 and |log_2_FC| > 0.5. Next, ggplot2 and pheatmap were utilized to show DE-miRNAs and DE-lncRNAs on heat maps and volcano plots, respectively. Subsequently, the mirDIP database was searched for miRNAs that interacted with prognostic genes. These miRNAs overlapped with DE-miRNAs, and the overlapped miRNAs were identified as key miRNAs. Next, lncRNAs interacting with key miRNAs were gained in the starbase database, and their intersections were taken with DE-lncRNAs, and the intersecting lncRNAs were labeled as key lncRNAs. Ultimately, the lncRNA-miRNA-mRNA network was created by prognostic genes, key lncRNAs, as well as key miRNAs.

### 2.16 Drug sensitivity analysis

According to GDSC database, using the pRRophetic software (v 0.5) (33), the IC50 of semi-inhibition of 138 chemotherapy/targeted therapy medications was determined for each BRCA patient. Between the two risk parts, the medication IC50 differences were contrasted. Next, the top 10 drugs with the highest significance were displayed using a box plot created by ggplot2.

### 2.17 Reverse transcription-quantitative polymerase chain reaction

A total of 10 tissue samples (5 normal and 5 BRCA) were acquired for PCR analysis. All participants were given informed consent. The study had the approval of the Xijing Hospital, The Fourth Military Medical University ethics committee (approval number:KY20162040-N-1). First, total RNA was extracted from the tissue samples through TRizol reagent, and then the concentration of total RNA was determined with the help of NanCChotometer N50. Next, RNA was reverse transcribed by SureScript First strand cDNA synthesis kit (Servicebio, China). PCR was carried out through CFX96 real-time PCR detection (CFX96; Bio-Rad, USA) systems with 40 cycles. The relative expression of genes was calculated by using the 2^-ΔΔCT^ method. The information sequences were shown in **Supplementary Table 4**, and GAPDH was used as an internal reference.

### 2.18 Statistical analysis

Bioinformatics analyses were undertaken utilizing the R programming language (v 4.2.2). A Wilcoxon test was utilized to compare the groups. When determining statistical significance, a p-value of less than 0.05 was used.

## 3 Results

### 3.1 Comparative analysis of immune infiltration between BRCA tissues and normal controls

**Figure 1:**
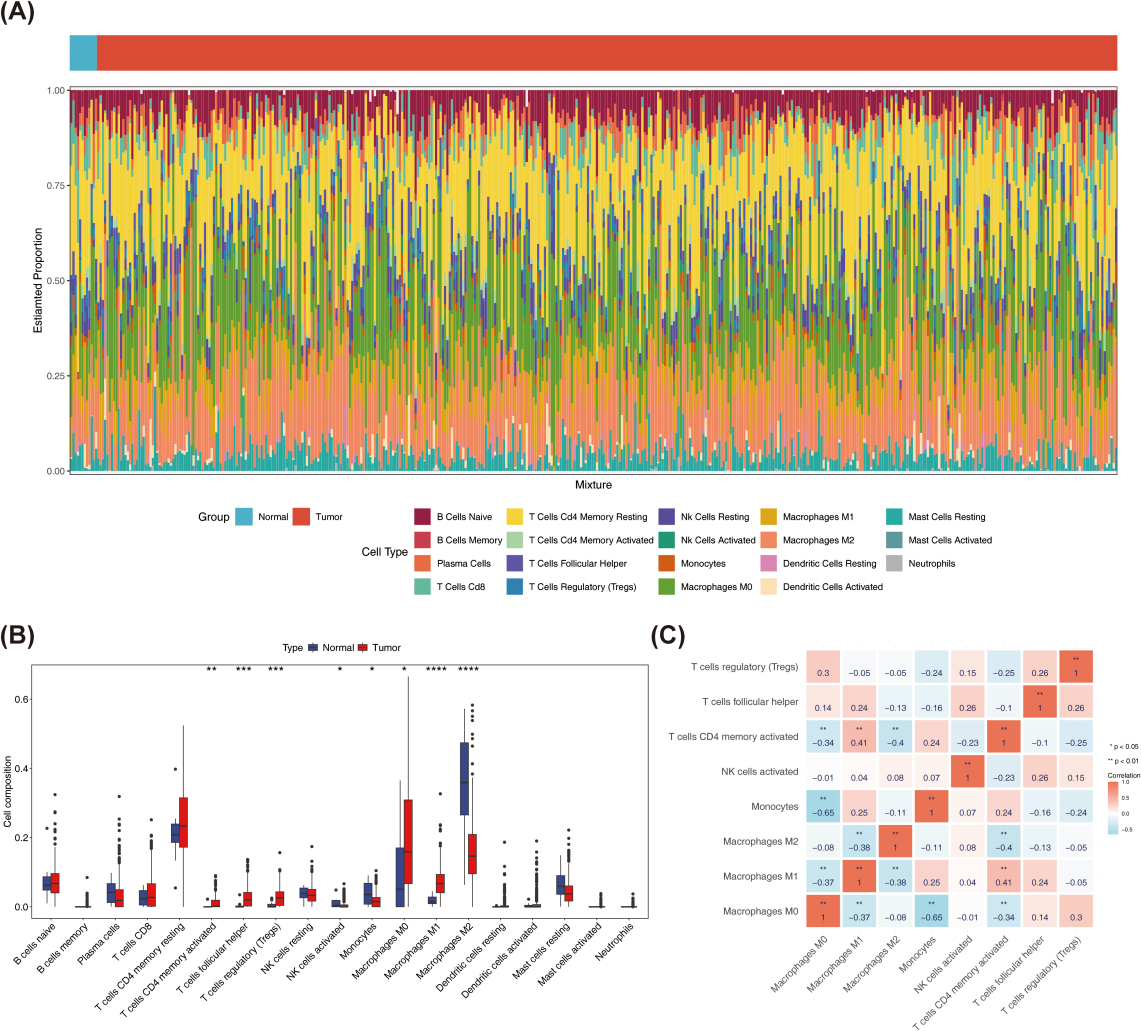
In this project, immune cells with a distribution ratio of 0 were removed when drawing, leaving 19 types of immune cells. **(A)** Immune cell ratio stacked bar chart in breast cancer and normal tissues. The x-axis represents samples, and the y-axis represents the overall ratio of different immune cells. **(B)** Spearman correlation analysis of the correlations between different immune cells in breast cancer and normal tissues. **(C)** Differences in the Correlation Heatmap of Immune Cells (* represents P < 0.05, ** represents P < 0.01; red represents positive correlation, blue represents negative). ***P < 0.001, ****P < 0.0001.

Comparative analysis of immune cell infiltration levels revealed distinct distribution patterns of 19 immune cell types between tumor and normal samples in TCGA-BRCA cohort (**Figure 1A**). Subsequently, eight immune cell types showed significant differences between tumor and normal samples. Among them, activated memory CD4^+^ T Cells, regulatory T cells (Tregs), T follicular helper cells, M0 macrophages, as well as M1 macrophages were more infiltrated in tumor samples, while M2 macrophages were more infiltrated in normal samples (**Figure 1B**). Additionally, Spearman correlation analysis revealed a significant negative correlation between monocytes and M0 macrophages (r = -0.65, p < 0.01), while M1 macrophages showed a positive significant relationship with activated memory CD4 T cells (r = 0.41, p < 0.01) (**Figure 1C**).

### 3.2 Screening of differentially expressed genes associated with T cell exhaustion and macrophage polarization

**Figure 2:**
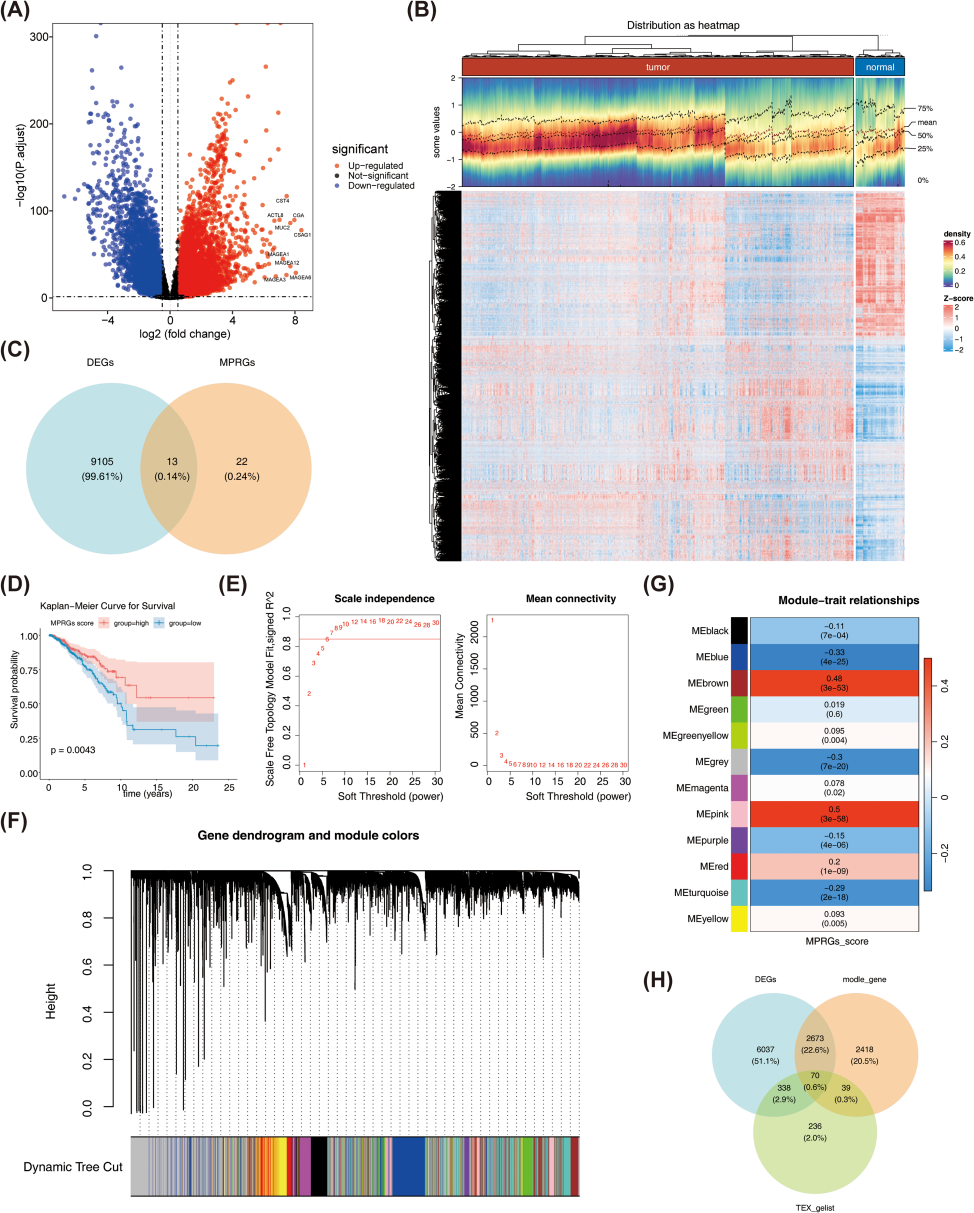
**(A)** Volcano plot of differentially expressed genes. Red dots indicate that their basal expression levels are up-regulated, blue dots indicate that their gene expression levels are down-regulated, and black indicate that these genes have no significant difference. **(B)** Heathop of differentially expressed genes. Each small square represents a gene, with higher expression levels shown in red and lower expression levels shown in blue. Each row represents the expression levels of gene across different samples, and each column represents the expression levels of all differentially expressed genes in a sample. **(C)** Venn diagram of differentially expressed MP-RG associated genes. **(D)** KM plot of MP-RGs score. **(E)** Scale-free soft-thresholding distribution: The x-axis represents the weight parameter power value, the y-axis of the left figure represents the scale-free fit index, i.e., the signed^2. The higher the coefficient of determination, the closer the network is to a scale-free distribution. The y-axis of the right figure represents the average of adjacency functions of all genes in the corresponding gene module. **(F)** Module clustering dendrogram: Genes were divided into various modules by hierarchical clustering, with different colors representing different modules, and gray representing the default for genes that cannot be classified any module. **(G)** Heatmap of the correlation between modules and clinical traits: The y-axis represents different modules, the x-axis represents MP-RG scores, and each block represents the correlation coefficient and significance P-value between a and a trait. **(H)** Candidate Gene Venn Diagram.

Following screening, 9,118 DEGs between BRCA and normal samples were found. Of these, up-regulated genes were 5,506, and down-regulated genes were 3,612 (**Figures 2A, B**). Then, 35 MPRGs were taken to intersect with 9,118 DEGs, and 13 DE-MPRGs were obtained (**Figure 2C**). Next, survival rates in the low-scoring part were relatively high (p = 0.0043) (**Figure 2D**). Subsequently, BRCA samples in TCGA-BRCA were clustered better with no outliers (**Supplementary Figure S1**). The optimal soft threshold (β) was chosen as 7 when R^2^ crossed the threshold 0.85 and mean connectivity also tended to 0 (**Figure 2E**). All genes were categorized into 11 modules (**Figure 2F**). Among them, MEblue, MEbrown, and MEgreen were selected as key modules and they contained genes 2588, 1724, and 888, respectively (**Figure 2G**). After combining the genes from the 3 modules, 5,200 key module genes were acquired. Finally, the key module genes, TEXRGs, and DEGs were crossed and 70 candidate genes were obtained (**Figure 2H**).

### 3.3 PPI network and functional analysis of candidate genes

**Figure 3:**
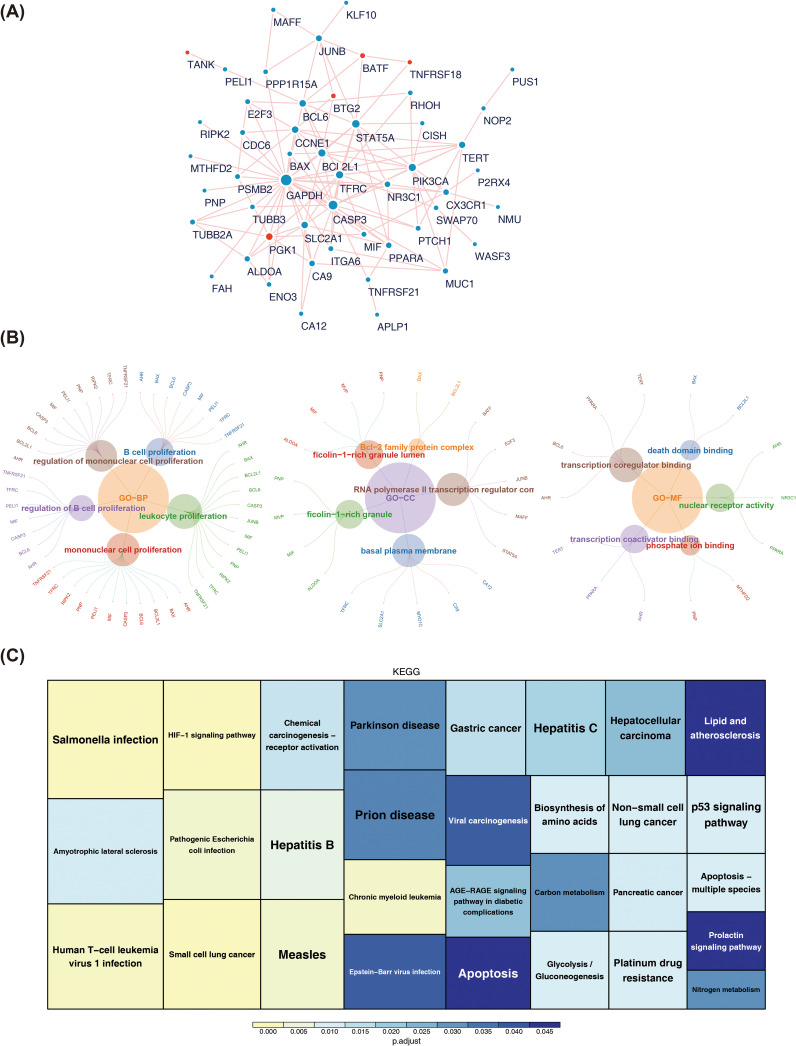
**(A)**PPI network among candidate genes. The red dots highlight the prognostic genes screened out by the 101 machine learning models in this study. **(B)** Tree map of GO enrichment of candidate genes. **(C)** KEGG pathway enrichment map of candidate genes.

The PPI network was created, in which it has 51 nodes and 128 edges. Among them, GAPDH and CASP3S had the most interactions with other genes, followed by TAT5A, PIK3CA, and BCL2L1 (**Figure 3A**). By enrichment analysis of the candidate genes, in biological process (BP), the candidate genes were significantly associated with B cell proliferation, regulation of B cell proliferation, mononuclear cell proliferation, etc. In molecular function (MF), the candidate genes were mainly enriched for entries such as death domain binding, transcription coregulator binding, nuclear receptor activity, transcription coactivator binding, phosphate ion binding, etc. In cellular component (CC), the Bcl−2 family protein complex, RNA polymerase II transcription regulator com, ficolin−1−rich granule, and basal plasma membrane were mainly enriched by candidate genes (**Figure 3B**). In KEGG analysis, candidate genes were mainly enriched for chronic myeloid leukemia, measles, and pathogenic Escherichia coli infection, among others (**Figure 3C**). These results suggested that the candidate genes were indeed involved in T cell related functions, which might also be related to macrophage polarization in BRCA patients.

### 3.4 Screening of prognostic biomarkers and construction of risk prediction models

**Figure 4:**
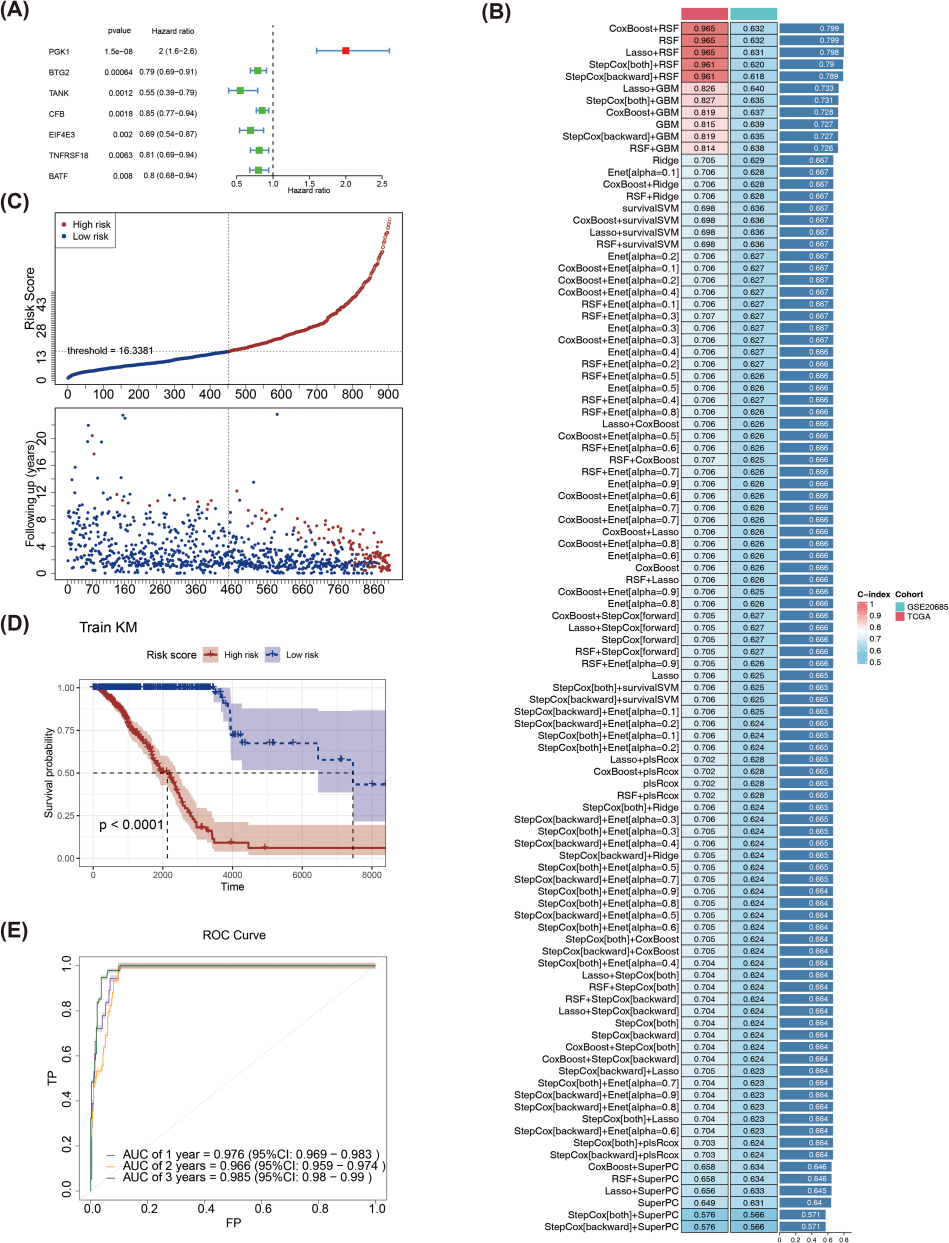
**(A)**Forest plot of univariate Cox regression analysis. **(B)** Evaluation of various machine learning models. **(C)** Risk curves for high and low risk groups in the training set. Red represents the high-risk group, blue represents the low-risk group, the x-axes of the two subplots are consistent, the risk scores patients increase from left to right, the y-axes are the risk score and survival time respectively, and the dotted line is the threshold for dividing high and low groups. **(D)** KM survival curve of the training set risk score. **(E)** The training set ROC curve evaluates the validity of the risk model.

By performing univariate Cox regression analysis on 70 candidate genes, 7 genes were selected with the names PGK1, BTG2, TANK, CFB, EIF4E3, TNFRSF18, and BATF (**Supplementary Table S3**). Then, forest plot was drawn for the 7 genes that passed the PH test, and it was observed that PGK1 (HR > 1, p < 0.05) was considered a risk factor, and BTG2, TANK, CFB, EIF4E3, TNFRSF18, as well as BATF were all protective factors (HR < 1, p < 0.05) (**Figure 4A**). The optimal model was then identified as the RSF algorithm (the highest C-index was 0.799), and PGK1, BTG2, TANK, CFB, EIF4E3, TNFRSF18, and BATF were screened as prognostic genes in the model (**Figure 4B**). Subsequently, the sample of BRCA patients was divided into high and low risk groups (the median risk score was 16.3381) (**Figure 4C**). The survival rate was relatively in low high-risk part (**Figure 4D**). Meanwhile, the risk model had a good predictability with area under the curve (AUC) of 0.976 (1 year), 0.985 (3 years) as well as 0.985 (5 years) (**Figure 4E**). Furthermore, risk model was validated through GSE20685. The risk curve, KM survival curve, as well as ROC curve analytical results were in agreement with the previously mentioned outcomes (**Supplementary Figures S2**–**S4**).

### 3.5 Risk score-clinical characteristic relationships

**Figure 5:**
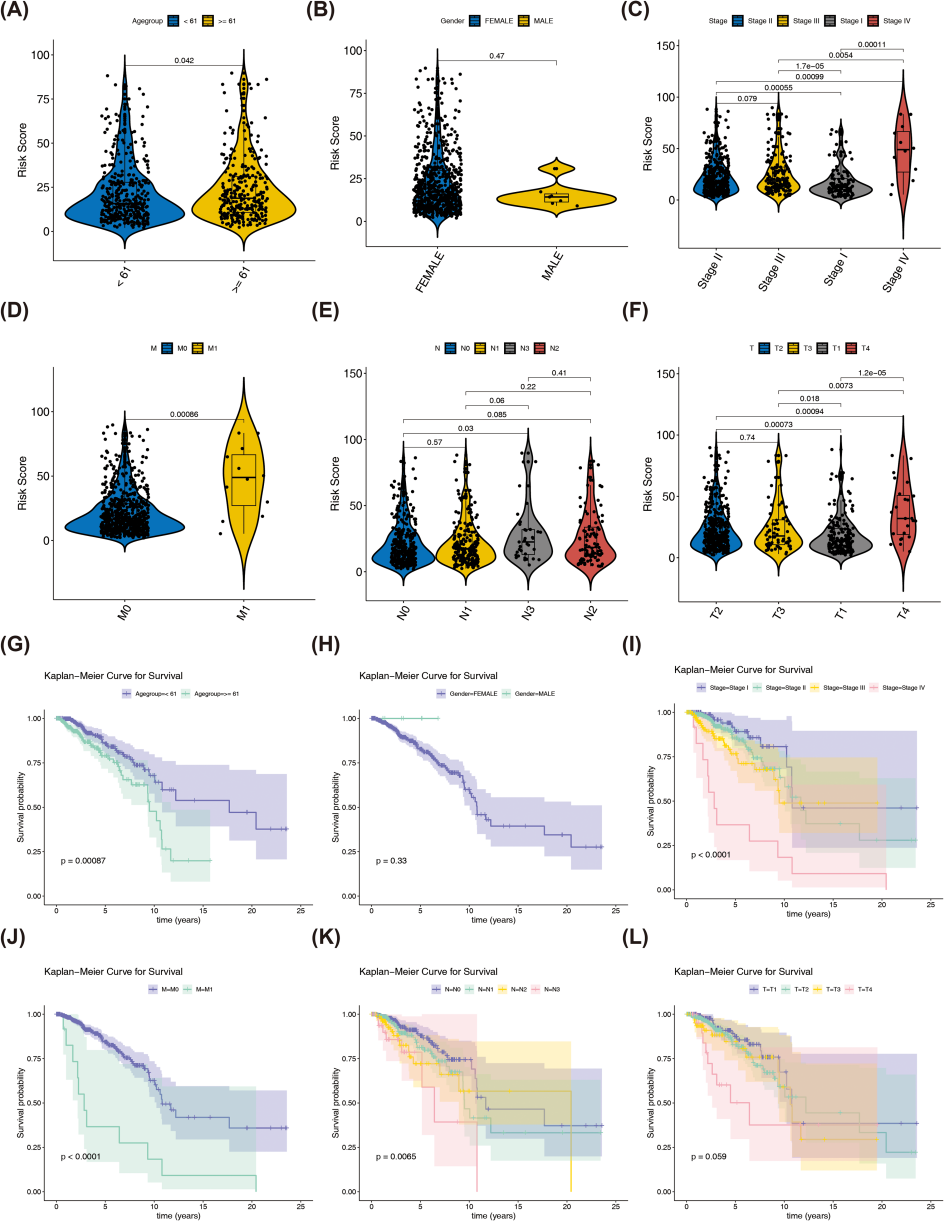
Differences in risk scores among different clinical characteristics and KM curves in different clinical characteristic groups. **(A)** The correlation between risk scores and age. **(B)** The correlation between risk scores and gender. **(C)** The correlation between risk scores and Stage group. **(D)** The correlation between risk scores and M group. **(E)** The correlation between risk scores and axillary lymph nodes. **(F)** The correlation between risk scores and tumor size **(G)** KM curves in age **(H)** KM curves in gender **(I)** KM curves in Stage group **(J)** KM curves in M group **(K)** KM curves in axillary lymph nodes **(L)** KM curves in tumor size.

Differences in risk scores were observed across several clinical characteristics, including age, tumor stage (excluding stage II–III), M stage, N0-N3 stages, and T stages (T1-T2, T1-T3, T1-T4, T2-T4, and T3-T4), but no significant differences were observed by gender (p < 0.05) (**Figures 5A-F**). Subsequently, by plotting KM survival curves for the above subtypes of clinical characteristics, survival differences were found in age, Stage, M, and N stages (p < 0.05) (**Figures 5G-L**).

### 3.6 Independent prognostic analysis and construction of nomogram

**Figure 6:**
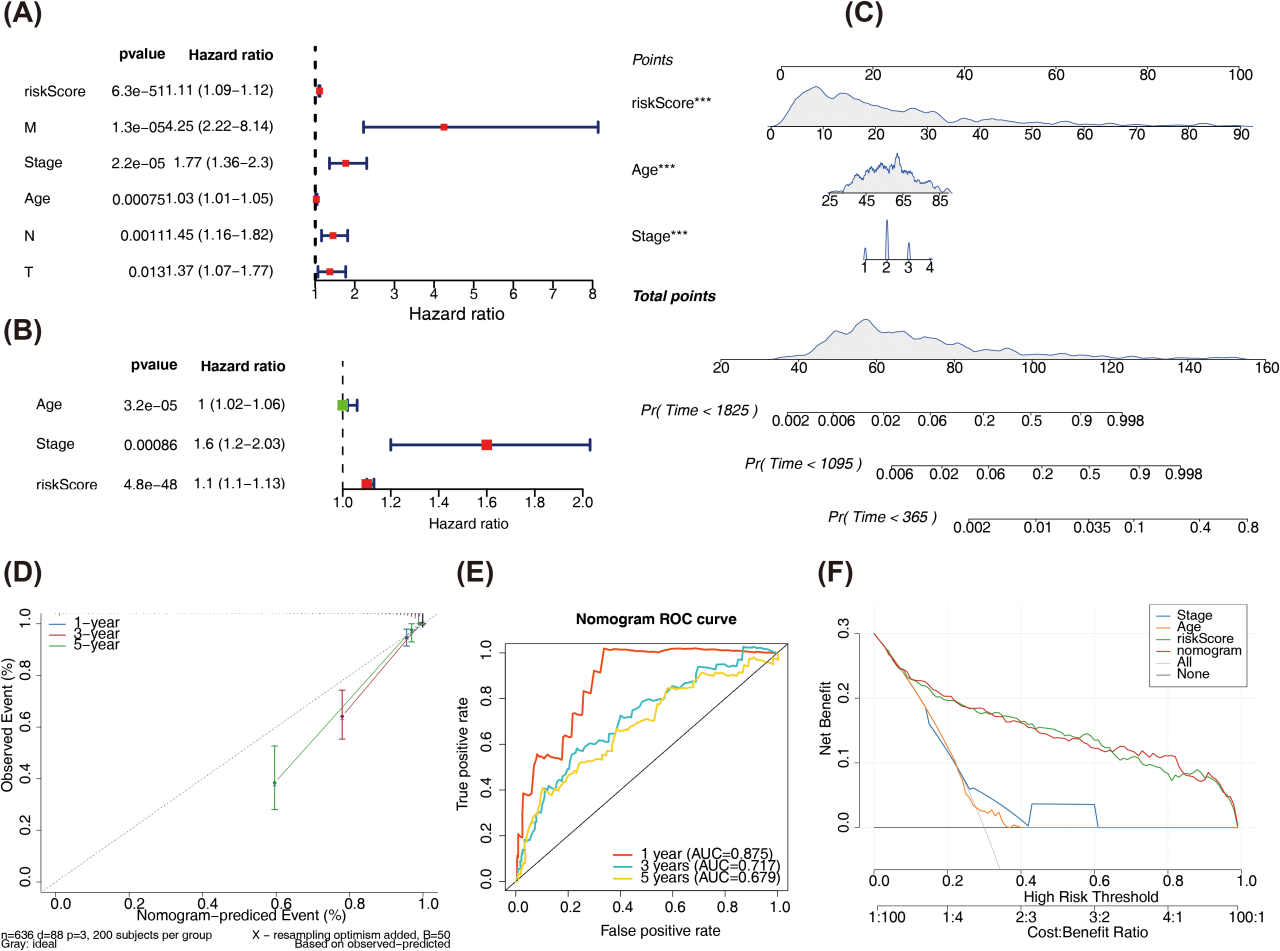
**(A)** Forest plot of univariate Cox regression results. **(B)** Forest plot of multivariate Cox regression results. The left side represents the factors and their corresponding P values and HR values; the red squares on the right indicate HR values greater than 1, green squares indicate HR values less than 1, and the lines on both sides of the squares represent the 95% confidence intervals for the HR values. **(C)** Nomogram predicts survival rate. The upper part shows the contribution of different clinical factors to the outcome variables, with each level of each factor assigned a score, as indicated by in the figure, representing the individual score for each clinical factor at different levels; the middle total score is the sum of the individual scores for each clinical factor; lower part shows the total point calculated and compared to the 1/3/5-year mortality probability for the patient. **(D)** Calibration curve results of the nomogram. **(E)** ROC curve of the nomogram. **(F)** Decision curve. The x-axis is the threshold probability: and the y-axis is the net benefit (NB) after subtracting the harm from the benefit. The slopes in the figure represent different clinical diagnostic models.

Age, risk score, stage, T, N, as well as M were the six outcomes of the independent prognosis of risk score and clinical characteristics that were found to be significant (HR > 1, p < 0.05). The PH assumption test was passed by all of them (**Figure 6A**, **Supplementary Figure S5**). Next, age, stage, as well as risk score were found to be significant independent prognostic variables in a multivariate Cox analysis (**Figure 6B**). A nomogram was constructed based on these variables, as shown in **Figure 6C**. Calibration curves indicated that the nomogram’s predictions were most accurate at 1 year, with slopes of 0.9459, 0.6339, and 0.3766 at 1, 3, and 5 years, respectively (**Figure 6D**). The AUC values were 0.875 (1 year) and 0.717 (3years) (**Figure 6E**). Moreover, at different threshold probabilities, the net benefit value of the DCA curve exceeded the separate independent prognostic factors and treat-all strategy (**Figure 6F**).

### 3.7 The immune landscape and genomic variation characteristics between the high- and low-risk groups

**Figure 7:**
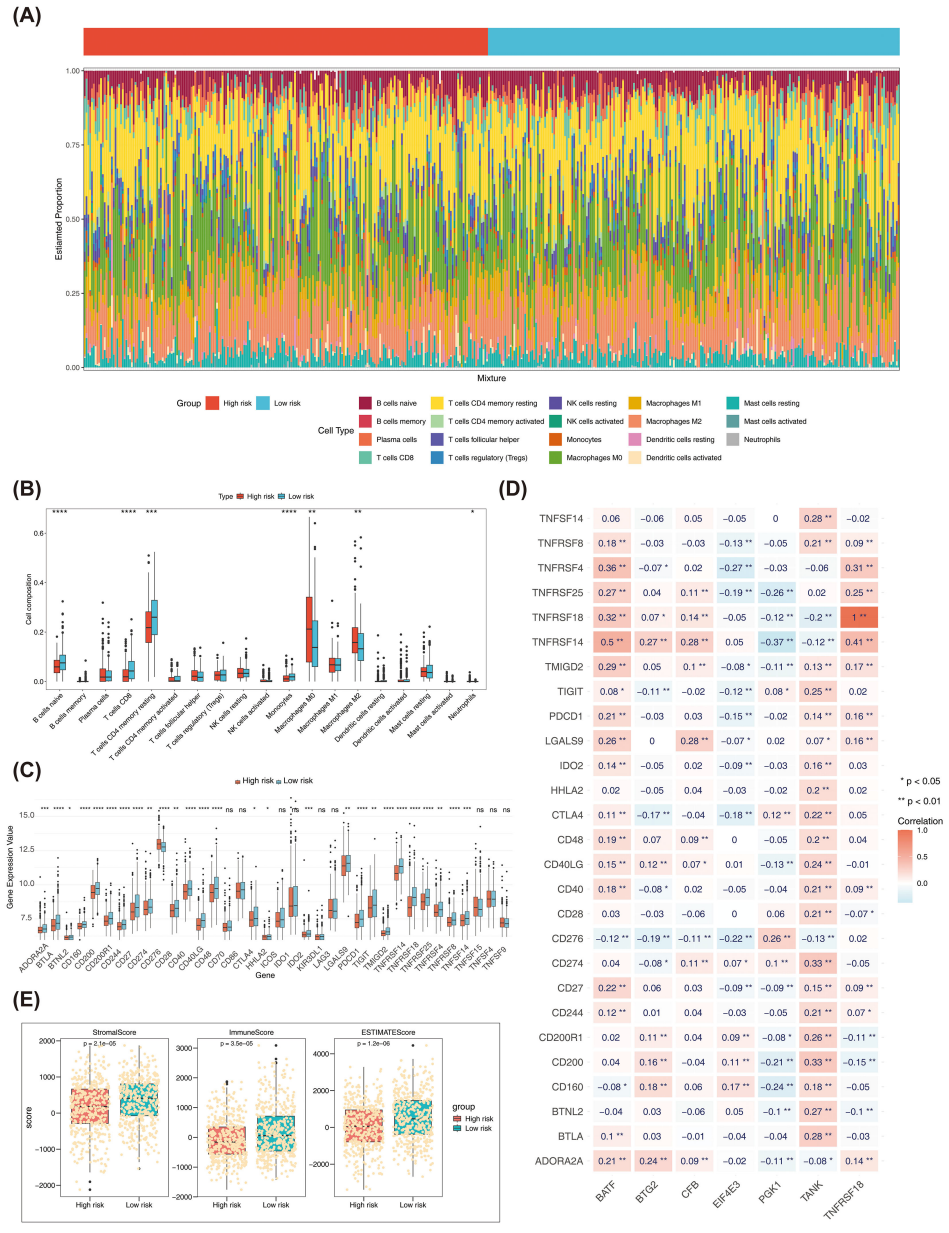
**(A)** Bar plots of immune cell infiltration in high and low risk groups. **(B)** Box plot of the proportion of immune cells in high and low risk groups. * represents P < 0.05, ** represents P < 0.01, *** represents P < 0.001 **** represents P < 0.0001. **(C)** Expression differences of ICI genes between high and low risk groups. The x-axis is the risk score, and the y-axis is the expression value of different immune checkpoints. **(D)** Heatmap of the correlation between ICI genes and prognostic genes. **(E)** Differences in immune score, stromal score, and estimate score between high- and low-risk groups in TCGA-BRCA. **(F)** Differences in TIDE, dysfunction, CD274, and exclusion between high and low risk groups in TCGA-BRCA. ns, no significant.

**Figure 7A** demonstrated the infiltration of 19 distinct immune cell types in the high- and low-risk groups. Seven immune cells (naive B cells and CD8 T cells) were shown to differ significantly (p < 0.05). Samples from the high-risk group showed larger infiltration abundances of M0 macrophages and M2 macrophages, whereas samples from the low-risk groups showed higher infiltration abundances of CD8 T cells and resting memory CD4^+^T cells (**Figure 7B**). Among the 36 ICIs, 27 showed significant differences (p < 0.05) between the two groups (**Figure 7C**). Positive connections were identified between BATF and TNFSF18 with TNFSF14 and TNFSF4, and between TANK and CD200 and CD274 (r > 0.3, p < 0.05) (**Figure 7D**). There were significant differences in the estimated score, stromal score, as well as immunization score between two risk group (**Figure 7E**). Additionally, there were notable distinctions between the two risk groups for TIDE, dysfunction, CD274, as well as exclusion between the two risk groups (**Figure 7F**).

**Figure 8:**
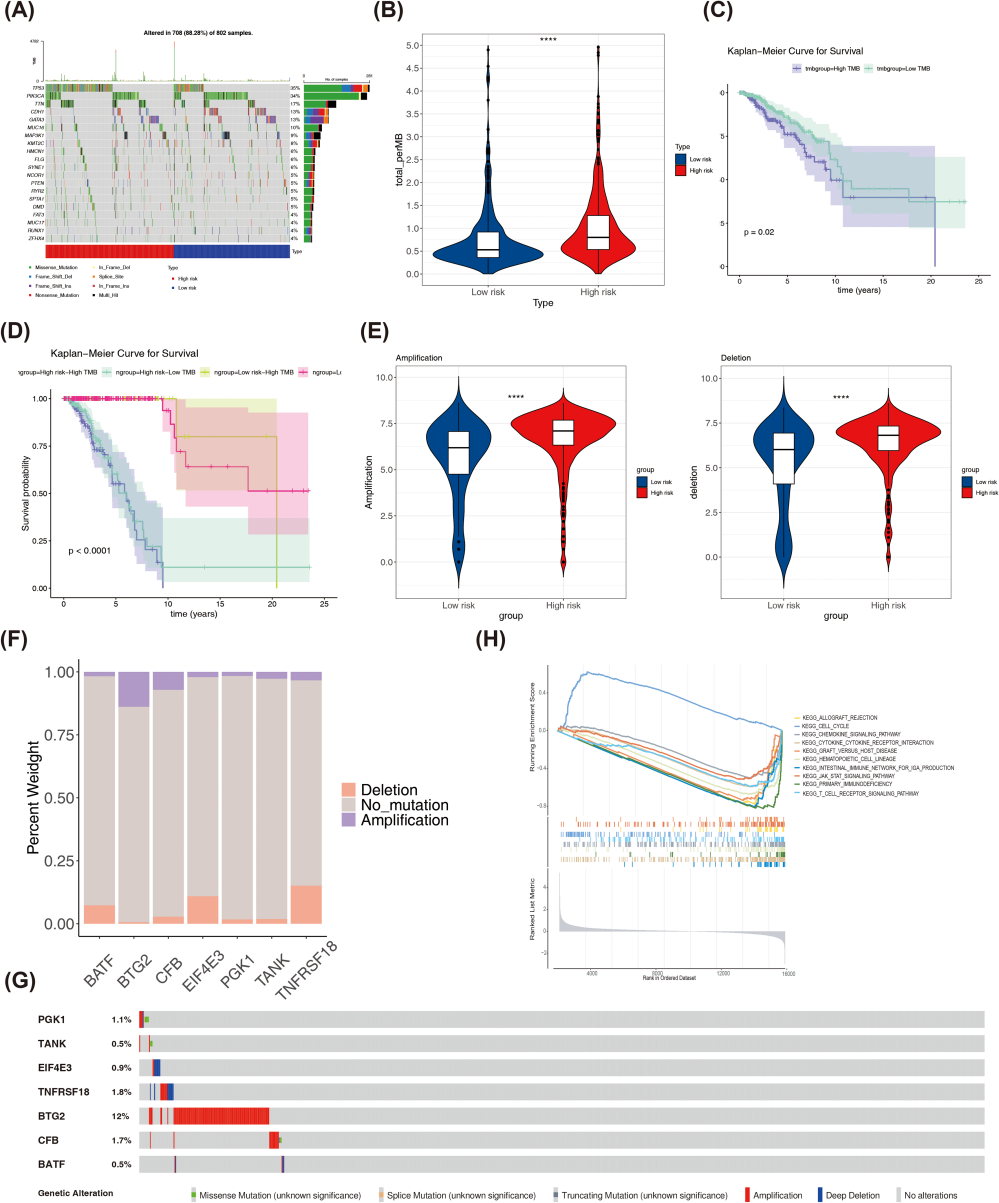
**(A)**The top 20 genes with the highest mutation frequencies in high and low risk groups. **(B)** Differences in TMB scores between high and low risk groups. **(C)** K-M curves of high and low TMB score groups. **(D)** K-M curves of H-TMB+high risk, H-TMB+low risk, L-TMB+high risk and L-TMB+ low risk patients. **(E)** Differences in copy number amplifications and deletions between high and low risk groups. **(F)** The amplification copy number and deletion copy number of prognostic genes in the TCGA-BRCA dataset. **(G)** The mutation frequency of each prognostic biomarker. **(H)** GSEA enrichment ridge plot. ****P < 0.0001.

Based on the TCGA-BRCA dataset, a mutation analysis was conducted. The top 20 genes with the highest mutation frequency in both risk categories were presented in **Figure 8A**. PIK3CA and CDH1 had high mutation frequencies in the low risk group, and TP53 and TTN had high mutation frequencies in the high risk group among the top 5 genes (**Supplementary Figure S6**). The high-risk part had higher TMB scores (**Figure 8B**). Patients had poorer survival rates in the high TMB group (**Figure 8C**). The survival rates were lower in high risk- high TMB as well as the high risk-low TMB group (**Figure 8D**). Both amplified copy number and deletion copy number were markedly different in two parts (**Figure 8E**). Meanwhile, BTG2 occurred with the highest frequency of copy number amplification in TCGA-BRCA, while TNFRSF18 and EIF4E3 occurred with the highest frequency of copy number deletion (**Figure 8F**). Moreover, by analyzing the mutation of prognostic genes in BRCA patients in TCGA-BRCA, it was found that among the copy number variations in 963 BRCA samples, 7 prognostic genes were mutated in a total of 165 samples (17%), of which BTG2 had the highest frequency of mutation in breast cancer, followed by TNFRSF18 and CFB (**Figure 8G**). Pathways significantly enriched in GSEA for the 7 prognostic genes were primary immunodeficiency, cytokine-cytokine receptor interaction, hematopoietic cell lineage, among others (**Figure 8H**). These findings suggested a potential association between these pathways and outcomes in BRCA patients.

### 3.8 The analysis of molecular regulatory networks and drug sensitivity

**Figure 9:**
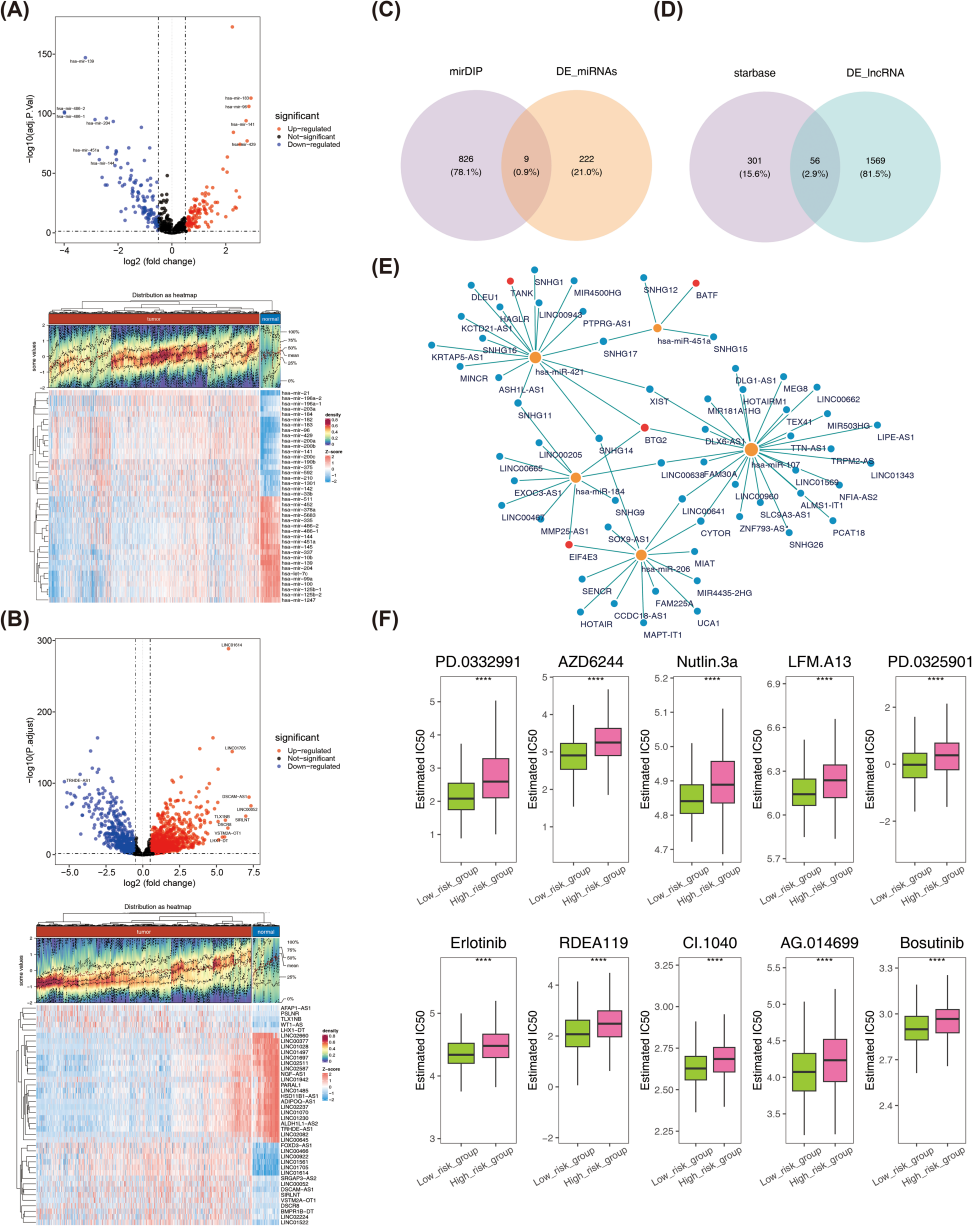
**(A)** Volcano plot and heatmap of differentially expressed miRNAs. **(B)** Volcano plot and heatmap of differentially expressed lncRNA. **(C)** Acquisition of key miRNAs. **(D)** Acquisition of key lncRNA. **(E)** lncRNA-miRNA-mRNA network. Blue dots represent lncRNAs, yellow dots represent miRNAs, and red dots represent prognostic genes. **(F)** Differences in IC50 values of 10 drugs between high- and low-risk groups. ****P < 0.0001.

A total of 231 DE-miRNAs were identified between BRCA and normal samples, with 115 miRNAs up-regulated and 116 miRNAs down-regulated in disease samples (**Figure 9A**). Similarly, 1,625 DE-lncRNAs were selected between BRCA and normal samples, with up- and down-regulation of 1,015 and 610, respectively (**Figure 9B**). The 9 key miRNAs were obtained by taking the intersection of DE-miRNAs and predicted miRNAs (**Figure 9C**). Similarly, 56 key lncRNAs were identified through overlapping DE-lncRNAs and predicted lncRNAs (**Figure 9D**). Finally, a lncRNA-miRNA-mRNA network containing 65 nodes (4 prognostic genes, 9 miRNAs, and 56 lncRNAs) and 70 edges was established. Among the interactions were SNHG17-hsa-miR-421-BTG2, UCA1-hsa-miR-206-EIF4E3, SNHG11-hsa- miR-421-TANK, among others (**Figure 9E**). Furthermore, the half maximal inhibitory concentration (IC50) of semi-inhibitory rates to 138 chemotherapy/targeted therapy drugs was assessed for each patient in BRCA, and the specific results were shown in **Supplementary Table S2**. Subsequently, the IC50 of 84 drugs was found to be significantly different in the high- and low-risk parts (**Supplementary Table S3**). The top 10 drugs with the highest significance (p-values ranked from smallest to largest) were PD.0332991, AZD6244, Nutlin.3a, LFM.A13, PD.0325901, Erlotinib, RDEA119, CI.1040, AG.014699, and Bosutinib, and the IC50 for these 10 drugs was relatively low in the low-risk group (**Figure 9F**).

### 3.9 Validation of prognostic gene expression levels

**Figure 10:**
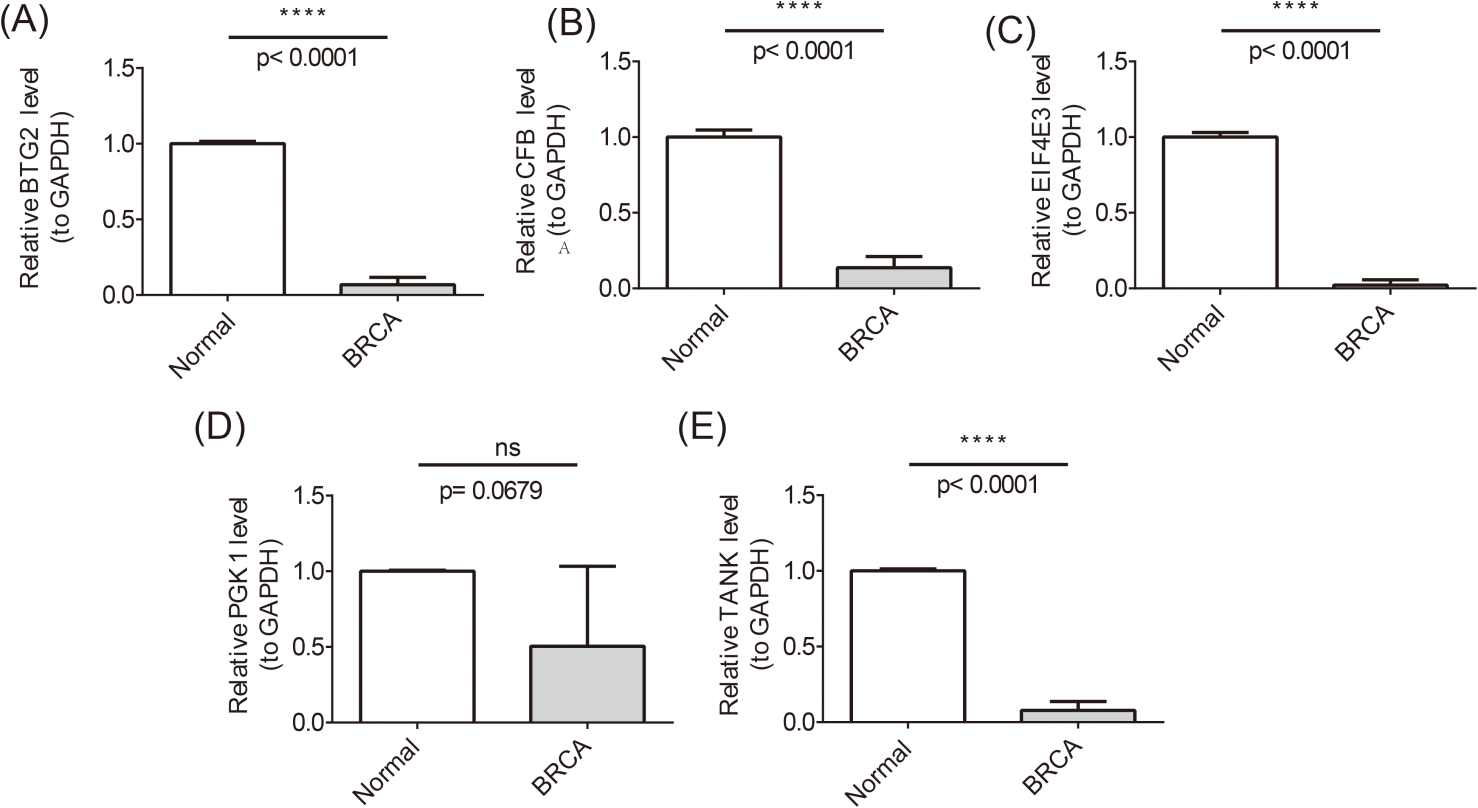
Differences in the expression levels of BTG2, TANK, CFB, and EIF4E3 in the normal and BRCA samples. ****P <0.0001; ns, no significant.

To ensure the reliability of the analyses, we used qRT-PCR experiments to validate the expression of prognostic genes. There were significant differences in the expression levels of BTG2, TANK, CFB, and EIF4E3 in the normal and BRCA samples. Specifically, BTG2, TANK, and EIF4E3 were significantly down-regulated in BRCA samples and were consistent with the results of the bioinformatics analyses, whereas CFB showed the opposite trend of the bioinformatics analyses, which may be due to the insufficient sample size or heterogeneity of the samples (**Figures 10A-E**).

## 4 Discussion

Metastatic disease is the leading cause of death in women with BRCA mutations and the current clinical treatment is surgery. However, the rate of disease recurrence after surgery is high and there is a significant degree of drug resistance. This severely limits the therapeutic efficacy of BRCA (34). TEX in breast tumors remains to be fully characterized (35). Macrophage accumulation is positively correlated with poor prognosis in BRCA patients (36). Previous studies have shown that tumor-associated macrophages present cancer cell antigens and induce T-cell dependent IRF8, which promotes tumor growth (37). It may be possible to further understand the mechanism of BRCA metastasis and reduce distant metastasis. However, the molecular mechanism between them is unclear. This study identified 7 prognostic genes (PGK1, BTG2, TANK, CFB, EIF4E3, TNFRSF18 and BATF) associated with T cell failure and macrophage polarization in BRCA by differential expression analysis, WGCNA, univariate Cox regression analysis, and machine learning algorithm. Moreover, a prognostic risk model was created to predict the survival of BRCA patients. In addition, analyses of immune infiltration, GSEA enrichment, molecular regulation, and drug sensitivity were performed. These findings provide valid targets for providing personalized treatment and prognostic interventions for BRCA patients.

The PGK1 gene is associated with the ability of cell lines to proliferate, migrate and invade (34). In BRCA patients, LINC00926 expression correlates negatively with PGK1 and positively with FOXO3A expression (38). PGK1 could be investigated not only as a biomarker, but also in combination with TP53/CDH1 for chemotherapy in BRCA (39). PGK1 may play an important role in BRCA. PGK1 exhibits complex and seemingly contradictory functional characteristics under different physiological and pathological conditions. Initially, it was identified as a tumor suppressor, capable of limiting tumor growth by reducing the disulfide bonds in plasmin outside the cell, thereby generating angiostatin. However, within the cell, it plays the opposite role (40). In terms of tumor metabolism, cancer cell proliferation and metastasis rely on energy supply from glycolysis (i.e., the Warburg effect), especially under hypoxic conditions in the tumor microenvironment (TME). Here, HIF-1α avoids degradation, dimerizes with HIF-1β, activates PGK1 expression, enhances the glycolysis pathway, and promotes the development of BRCA (41). Multiple studies (42, 43) have shown that PGK1 expression is significantly upregulated in various BRCA subtypes and is closely related to tumor malignancy and prognosis. Meanwhile, in the field of diabetic cardiomyopathy (DCM), research has found that PGK1 may play a promoting role by driving the polarization of M1 macrophages (44). However, when siPGK1 interference is applied, it can regulate the inflammatory response by inhibiting macrophage pyroptosis and M1 macrophage polarization (45). Overall, the dual nature of PGK1’s function is highlighted. It is highly likely that by regulating the glycolysis pathway and intervening in the polarization state of macrophages, PGK1 deeply participates in and influences the occurrence and development of BRCA. These findings suggest that intervention targeting PGK1 and its modifications may represent a potential therapeutic strategy for cancer treatment.

BTG2 is a member of the BTG/TOB family of anti-proliferative proteins. BTG2 could inhibit proliferation and migration and regulate cell cycle progression (46). High expression levels of BTG2 were reported to significantly affect BRCA patient survival (47). Additionally, BTG2 may play a role in the polarization of M2-type macrophages and the tumor microenvironment. Studies have shown that the inhibition of M2-type macrophage polarization can suppress the metastasis of BRCA. This suggests that inhibition of macrophage polarization and tumor cell proliferation and migration is important for BRCA (48). This is consistent with our findings, where the infiltration abundance of M2-type macrophages was higher in the high-risk group samples. Additionally, BTG2 is a key component of the pre-B cell differentiation program. Together with PRMT1, it forms the BTG2-PRMT1 module, which induces cell cycle arrest in pre-B cells (45). In this study, BTG2 is found to be lowly expressed in BRCA tissues, while B cell naive is highly expressed in the high-risk group. Therefore, BTG2 may play a role in the progression of BRCA by affecting macrophages cell proliferation and migration.

TBK1 is a serine/threonine kinase and a non-canonical member of the IKK family with multiple cellular functions in innate immunity, tumorigenesis and development. TBK1 is a key regulator of centrosome homeostasis and is required for mitotic progression in BRCA (49). Thus, TANK may function in BRCA by regulating the mitosis of BRCA cells.

Mutations in the CFB gene result in reduced activation of B cells, which in turn lead to changes in the tumor immune environment. This may play a role in the correlation between the presence of these mutations and unfavorable outcomes in breast and lung cancer (50). The subunits are regulated by factor D, which is associated with proliferation and differentiation of preactivated B lymphocytes, rapid expansion of peripheral blood monocytes, stimulation of lymphocyte follicle formation and erythrocyte lysis. Mutations in the CFB gene lead to reduced activation of B cells, which in turn leads to changes in the tumor’s immune environment. This may contribute to the observed association between the presence of these mutations and unfavorable outcomes in breast and lung cancer (51). Additionally, the expression of CFB is downregulated in clinical samples of lung adenocarcinoma (LUAD), while its overexpression can inhibit tumor growth, cell proliferation, and migration, and promote cell apoptosis and cell cycle arrest. CFB can also suppress tumor development by negatively regulating the Ras/MAPK signaling pathway. Knockdown of CFB may inhibit the NF-κB signaling pathway (52), thereby driving macrophage polarization towards the M2 phenotype (53), and also affect B cell activation (54). In our study, the expression level of CFB was significantly reduced in the BRCA group. In summary, CFB may influence the occurrence and development of BRCA by regulating macrophage polarization and B cell activation.

EIF4E3, a member of the EIF4E family of translation initiation factors, has been observed to interact with the 5′-cap structures of mRNA (55). Prior research has demonstrated that individuals exhibiting elevated EIF4E expression are more prone to recurrence and mortality compared to those displaying diminished eIF4E expression in patients with triple-negative BRCA (56). EIF4E plays a pivotal role in the protein complex known as the translation initiation factor. Individuals with elevated EIF4E expression are at an increased risk of recurrence and mortality compared to those with reduced EIF4E expression in triple-negative BRCA patients. EIF4E exerts a significant influence on the development of BRCA (57). In our study, the expression level of EIF4E3 was significantly decreased in the BRCA group, indicating that EIF4E3 may function as a tumor suppressor gene in breast cancer, and its low expression may be associated with the progression of the disease.

TNFRSF18 is a member of the tumor necrosis factor receptor (TNF) superfamily and plays a significant role in immune responses. It is involved in T cell co-stimulatory signaling and exerts crucial functions in protective immunity, inflammation, autoimmune diseases, and cancer immunotherapy. In the METABRIC cohort, patients with high expression of TNFRSF18, which encodes GITR, had significantly improved survival compared to those with low expression. The addition of local RT or an anti-TNFRSF18 agonist to PD-L1 blockade did not result in a significant increase in efficacy compared to PD-L1 blockade alone. However, the combination of both with PD-L1 blockade showed a significant reduction in tumor growth and lung metastases (58). This suggests that the GITR agonist may enhance T cell activation and antitumor immune responses, while radiation therapy further promotes the release of tumor antigens and the infiltration of immune cells, thereby synergizing with PD-L1 blockade. In our study, the infiltration abundance of T cells was higher in the low-risk group samples, indicating that patients in the low-risk group may have a stronger antitumor immune microenvironment, which is associated with better prognosis.

BATF is a member of the AP-1/ATF superfamily of proteins. BATF inhibits Ras and Fos-mediated cellular transformation by acting as a negative regulator of AP-1-mediated transcription (59, 60). Studies have shown that the deletion of BATF can enhance the antitumor activity of CAR-T cells, particularly in combating T cell exhaustion. In various types of CAR-T cells and mouse OT-1 cells, the absence of BATF rendered T cells more resistant to exhaustion and endowed them with a stronger capacity for tumor eradication. These investigations have unveiled BATF as a key regulator of T cell function, with its deletion potentially enhancing T cell persistence and antitumor activity, thereby offering new strategies for cancer immunotherapy. In our study, the infiltration abundance of T cells was higher in samples from the low-risk group, which may be associated with stronger T cell function and lower levels of exhaustion in patients of the low-risk group, thus correlating with better clinical outcomes. Calycosin has been demonstrated to inhibit the progression of BRCA cells by suppressing epithelial-to-mesenchymal transition (EMT) via the BATF/TGFβ1 pathway (61).

This study constructed a clinical risk prediction model by aggregating the results of multivariate Cox regression analysis. The results indicate that high-risk patients have a significantly worse prognosis, with a higher risk score being positively associated with more aggressive clinical characteristics. DCA is a well-established method for the evaluation of nomograms, which have been tailored to meet the practical requirements of clinical decision making (62). Furthermore, ROC curve, which assesses the validity of the risk model, demonstrated that AUC values at 1, 3, and 5 years were all greater than 0.9 (0.976 at 1 year, 0.985 at 3 years, and 0.985 at 5 years). This performance exceeds the moderate accuracy of established models such as the Breast Cancer Risk Assessment Tool (BCRAT) and the Breast Cancer Surveillance Consortium (BCSC), which have a maximum AUC of 0.71 (63). Further evidence that t-cell exhaustion and macrophage polarization are common genes that predict disease is good and can provide a valuable reference tool for clinical decision-making. This provides a distinctive viewpoint with regard to the prediction of cancer risk. However, this model was constructed solely through bioinformatics analysis, and its reliability and clinical applicability still require further validation through experimental studies and clinical data. Therefore, we plan to systematically validate the model’s key molecular mechanisms and predictive performance by integrating *in vitro* experiments, animal models, and clinical samples in future research.

The analysis of immune infiltration revealed significant differences in seven distinct immune cell types between the high- and low-risk groups. These included naive B cells, CD4 T cells, CD8 T cells, monocytes, neutrophils, M0 and M2 macrophages. Eribulin has been observed to promote CD8^+^ T cell proliferation, repress effector T cell differentiation, and harness T cell-mediated anti-tumor effects. These mechanisms may serve as a potential indicator of eribulin’s ability to enhance the immunological status of tumor-bearing hosts. Studies have demonstrated that the combination of CD8^+^ T cell functional status assessment and immunosuppressive factor analysis can provide clinically relevant information for different BRCA subtypes (64). Programmed death-ligand 1 (PD-L1), encoded by the CD274 gene, is expressed on the surface of various cells within the TME. Immune checkpoint inhibition therapy, particularly PD-1/PD-L1 blockade, has demonstrated considerable promise in recent years and represents a promising area of research. It has been demonstrated that macrophages of the M2 phenotype promote the expression of PD-L1 in triple-negative BRCA cells through the secretion of CXCL1 (65). Furthermore, it is generally accepted that plasma cells, monocytes, resting dendritic cells, resting mast cells and resting memory CD4^+^ T cells are associated with a favorable prognosis (66). A detrimental prognosis was associated with the presence of M0, M1, and M2 macrophages; activated dendritic cells; activated mast cells; regulatory T cells; T follicular helper cells; and neutrophils (67).

The GSEA revealed that pathways significantly enriched for the seven prognostic genes included primary immunodeficiency, cytokine receptor interaction, and hematopoietic cell lineage, among others. Primary immunodeficiency diseases encompass a range of genetic disorders that affect various components of innate and adaptive immune responses (68). Xia (69) et al. found that the expression of primary immunodeficiency genes has an extremely important impact on the development, prognosis, tumor environment, and treatment response of triple-negative BRCA patients. Cytokine-receptor interactions play a crucial role in the immune system, not only regulating allergic inflammation and immune responses but also exerting key functions in tumor immunity. For instance, interleukin (IL)-4 and IL-13 regulate the functions and gene expression of various immune cells by binding to specific receptors, thereby influencing allergic reactions and the health of the immune system. Moreover, the signaling of IL-2 through its receptor plays an important role in tumor immunity, promoting the activation, differentiation, and functional restoration of T cells, and providing potential strategies for tumor immunotherapy. These studies demonstrate that cytokine-receptor interactions have broad applications in immune regulation and disease treatment. The formation and development of the hematopoietic cell lineage is a continuous process rather than being hierarchically organized through discrete progenitor populations. Specifically, blood formation occurs through the gradual differentiation of hematopoietic stem cells (HSCs), with stepwise acquisition of lineage bias along multiple trajectories, rather than through the traditional progenitor hierarchy model. The self-renewal capacity of multipotent HSCs supports hematopoietic system homeostasis throughout life and underpins the therapeutic potential of clinical HSC transplantation (70).These findings suggested a potential association between primary immunodeficiency, cytokine-cytokine receptor interaction and outcomes in BRCA patients. This further suggests that the seven prognostic genes may play a role in BRCA by participating in primary immunodeficiency and cytokine-cytokine receptor interaction.

In this study, the IC50 of 84 drugs was found to be significantly different in the high- and low-risk parts. The top 10 most significantly different drugs included PD.0332991, AZD6244, Nutlin-3a, LFM.A13, PD.0325901, Erlotinib, RDEA119, CI.1040, AG.014699, and Bosutinib, with the IC50 for these drugs being relatively low in the low-risk group. Nutlin.3a, the P53 activator is critical in preventing tumorigenesis, and P53-activating drugs, may have unexpected effects on macrophage function (71, 72). The administration of bosutinib has been identified as a promising radiosensitizer, as it markedly reduces the dosage required for both the drug and ionizing radiation (73). This may be associated with a reduction in treatment-associated adverse reactions. The present study suggests that bosutinib may be an effective potential radiosensitizer in the treatment of BRCA. Furthermore, experimental evidence corroborated these findings, indicating that eIF4G1 silencing led to the downregulation of DDR proteins, thereby enhancing radiosensitivity in BRCA cells. Therefore, bosutinib may be a promising radiosensitizing agent for the treatment of BRCA (73). These results suggest that the drug may stimulate macrophages to fight drug-resistant bacteria, thereby boosting innate immunity (74, 75). Erlotinib is a reversible, small molecule ATP-competitive EGFR inhibitor that is primarily used in the treatment of non-small cell lung cancer (76). Erlotinib binds to the ATP-binding pocket of EGFR, thereby preventing phosphorylation and subsequent activation of downstream cell cycle progression, proliferation, and angiogenesis signaling pathways. In a subset of BRCA patients, erlotinib demonstrated efficacy in clinical trials (77).

This study was based on an analysis of publicly available databases. Following a series of screening procedures, a total of seven prognostic genes were identified. A risk model was constructed, and immune infiltration analysis, function enrichment analysis and molecular regulatory network analysis were carried out in the high- and low-risk groups. The methodology employing bioinformatics analysis to screen breast cancer prognostic genes demonstrates multifaceted advantages compared to traditional experimental approaches. Firstly, bioinformatics analysis enables efficient processing of large-scale genomic, transcriptomic, and clinical data, rapidly identifying candidate genes associated with breast cancer prognosis. This significantly reduces research timelines and decreases preliminary screening costs. By integrating data from public databases, researchers can fully utilize existing resources to avoid redundant experiments while elucidating intricate gene regulatory networks and functional pathways, thereby offering a more comprehensive perspective for investigating breast cancer’s molecular mechanisms. In contrast, conventional experimental methods typically demand substantial time, human resources, and financial investment, yet struggle to comprehensively capture gene-gene interactions and regulatory relationships. Additionally, compared with previous BRCA biomarker studies (78), we employed a more comprehensive machine learning strategy (10 algorithms, 101 combinations) to optimize model construction, which significantly enhanced predictive performance (the 1-, 3-, and 5-year AUC of the risk model in both the training and validation sets were all > 0.6). Moreover, we not only conducted independent prognostic analyses but also further constructed a clinically useful nomogram. The decision curve analysis (DCA) curve confirmed its good clinical decision utility. The biological value of the biomarkers was also validated through multidimensional analysis, including prediction of immune checkpoint (ICI) response, dissection of lncRNA-miRNA-mRNA regulatory networks, and drug sensitivity analysis. Therefore, this study provides valuable insights into the molecular mechanisms of macrophage polarization and TEX-related prognostic genes in BRCA, which could enhance the precision of early BRCA prediction.

However, there are some limitations to this study. Further experimental validation, an expanded sample size, and a combined molecular typing approach are necessary to further investigate the prognosis. The quality and completeness of the database used in this study may influence the accuracy of the analytical results. Incomplete or biased data could potentially affect the study findings. In terms of drug sensitivity analysis, due to limitations in data acquisition, we were unable to adjust for the impact of potential confounding factors on IC50. Secondly, the key genes identified through bioinformatics screening lack validation from laboratory experiments and clinical data. Regarding molecular mechanism validation, the predicted lncRNA-miRNA-mRNA interactions and copy number alterations lack experimental validation. Sole reliance on computational analyses may not fully reflect the complexities of biological systems. In future studies, we plan to integrate experimental and clinical research to further validate the functional roles of these key genes and their potential involvement in disease mechanisms.

Nevertheless, our research provides novel insights into the intricate relationships between genes, proteins, and other biomolecules, which may contribute to a deeper understanding of disease pathogenesis and facilitate drug discovery. These findings offer potential theoretical foundations for advancing precision medicine and serve as a reference for exploring personalized therapeutic and preventive strategies.

## Data Availability

The datasets presented in this study can be found in online repositories. The names of the repository/repositories and accession number(s) can be found in the article/**Supplementary Material**.
