# Supplementary material for: Identification and validation of prognostic genes associated with T-cell exhaustion and macrophage polarization in breast cancer
Source: Front Endocrinol (Lausanne). 2025 May 27;16:1556496. doi: 10.3389/fendo.2025.1556496 (PMC12148895; doi:10.3389/fendo.2025.1556496)
Supplement: Supplementary file 6 [file DataSheet1.docx]

**Identification and validation of prognostic genes associated with T-cell exhaustion and macrophage polarization in breast cancer**


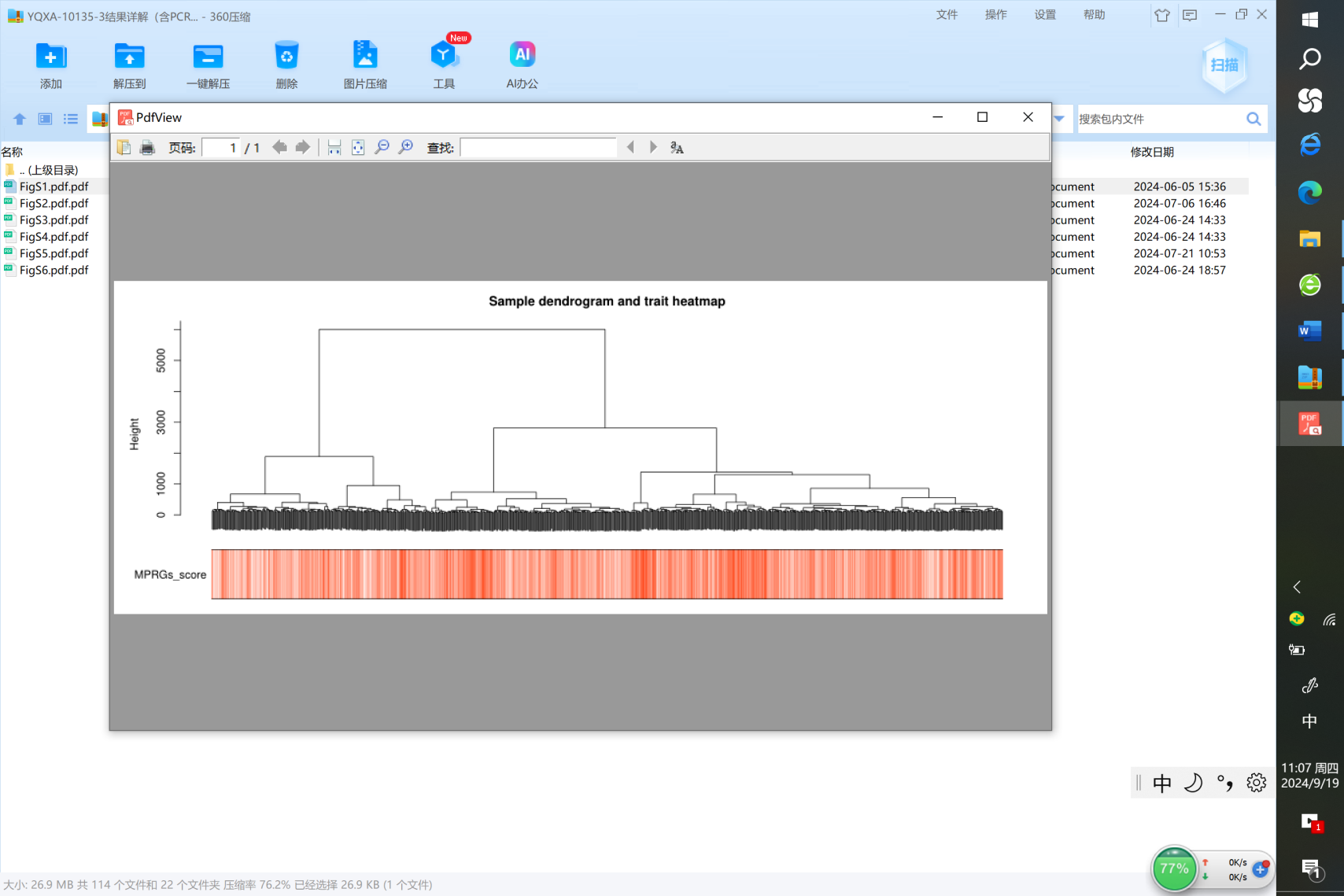


**Fig. S1** Sample clustering and shape heat map（The top half of the graph is the sample clustering case, the branches represent the samples, the vertical coordinates represent the height of the hierarchical clustering, and the bottom half is the traits, corresponding to the branches, and the colors represent the MP-RGs scores）


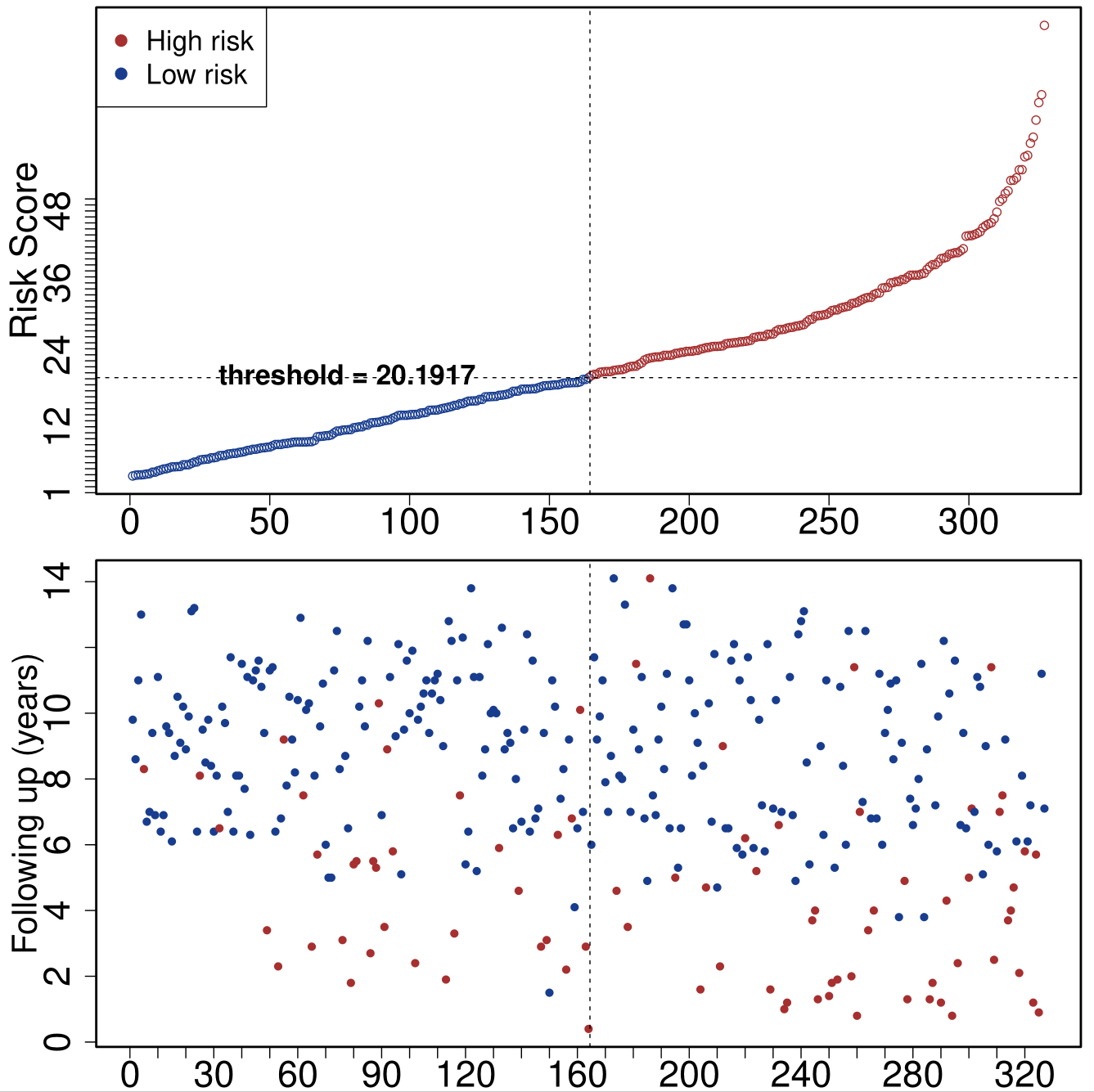


**Fig. S2** Risk curves and scatter plots of high and low risk groups in GEO datasets


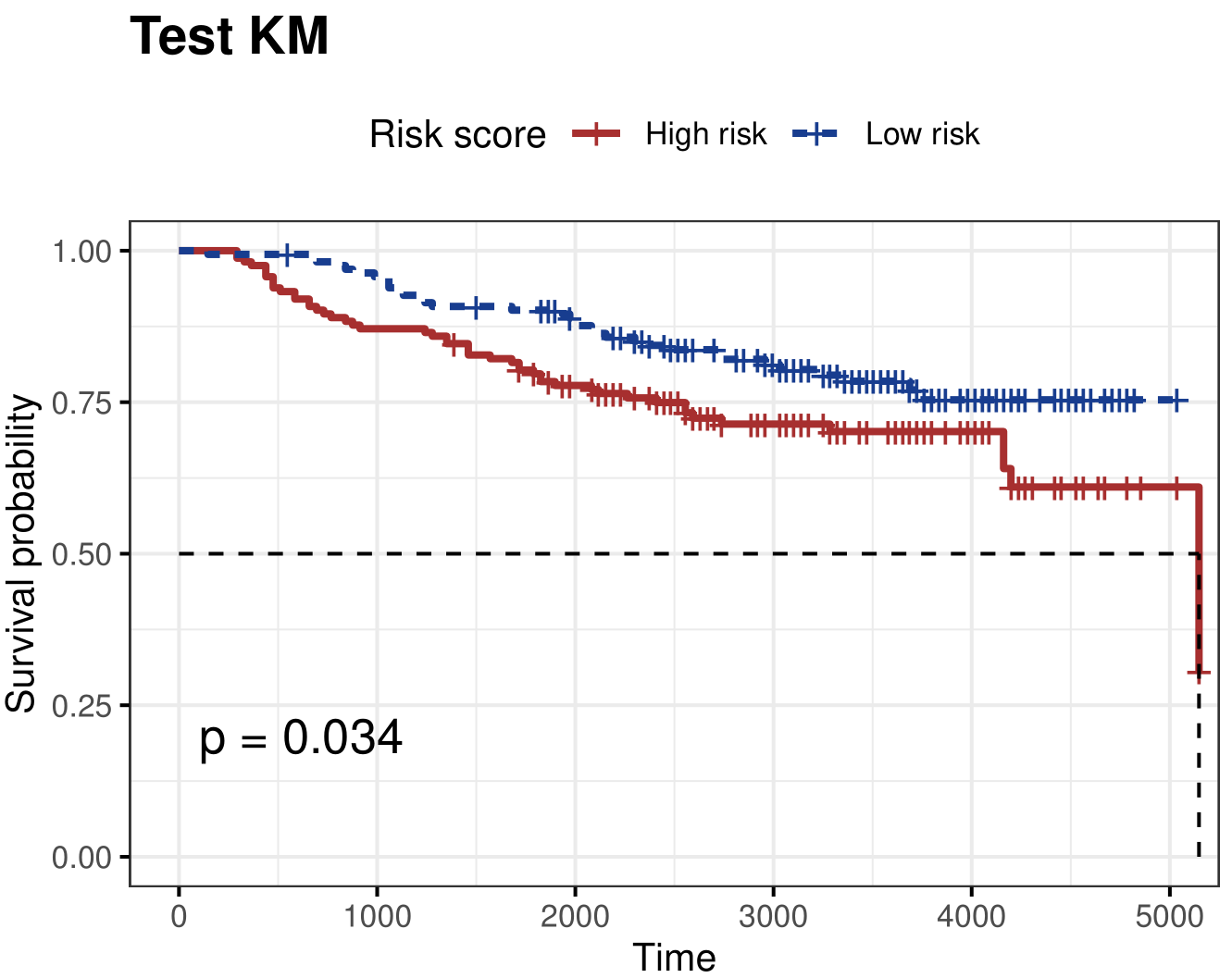


Fig. S3 Survival curves of high and low risk groups in GEO datasets. The y-axis of the figure represents the survival rate, and the x-axis represents the overall survival time. Red represents the high-risk group, and represents the low-risk group.


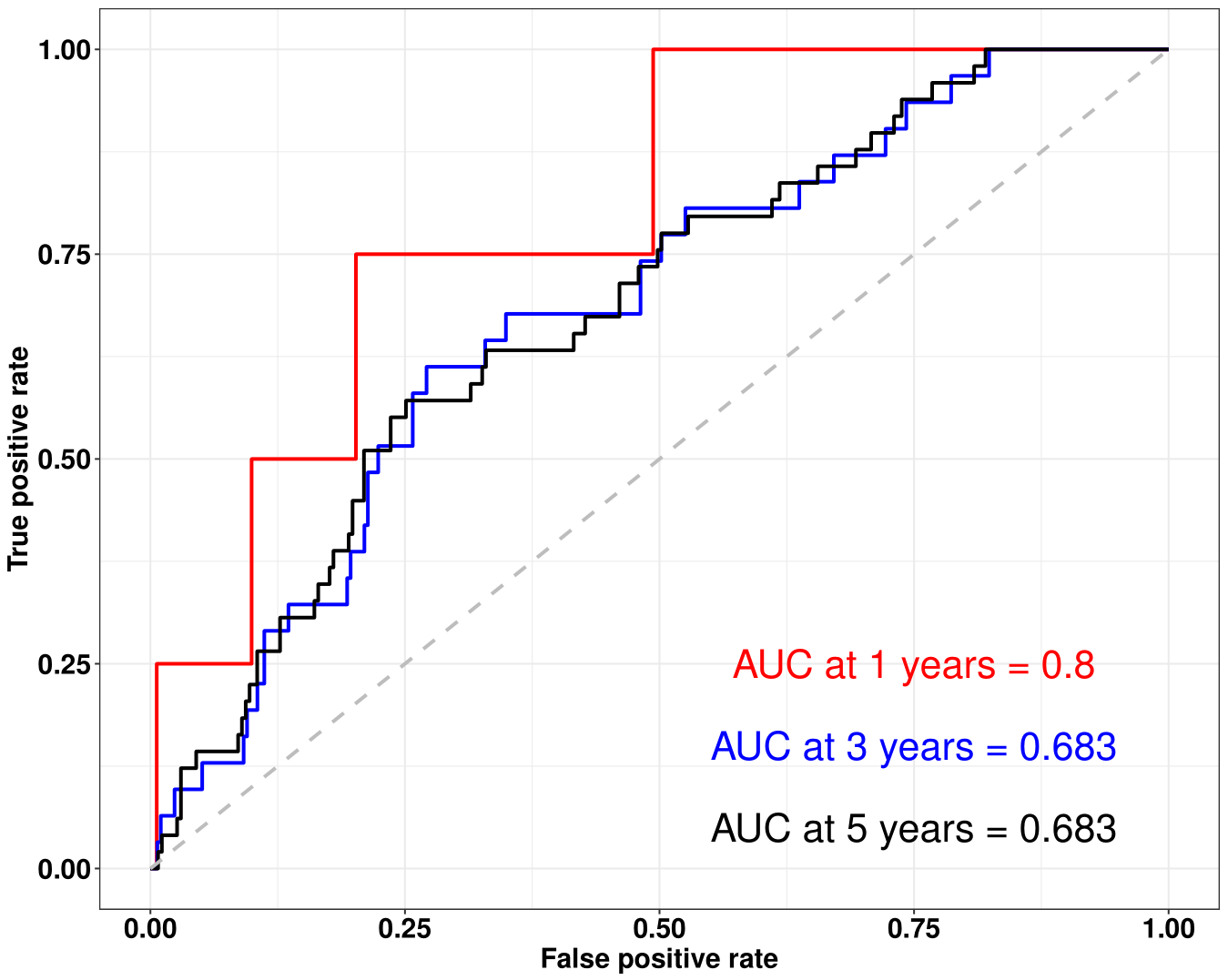


**Fig. S4** ROC curves for 1/3/5-year survival in the GEO datasets.


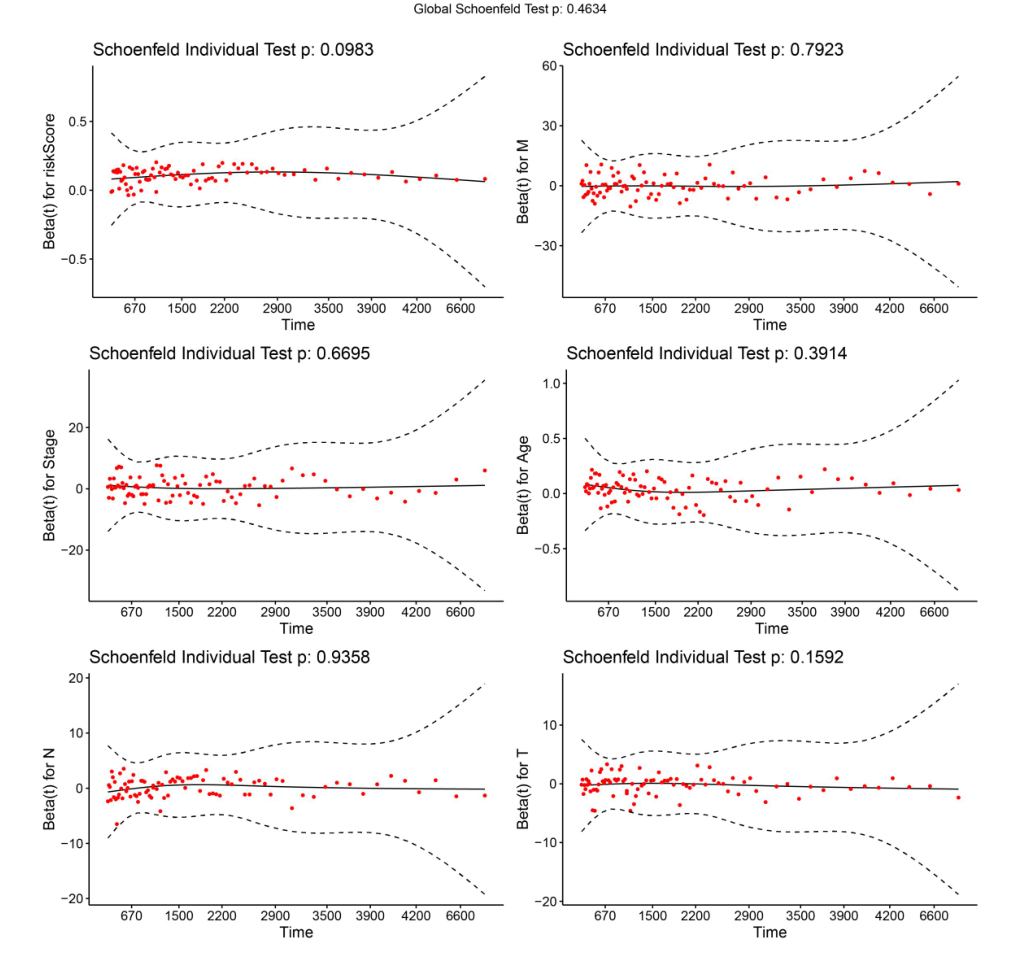


**Fig. S5** Perform PH assumption test on factors with univariate p-value < 0.05.


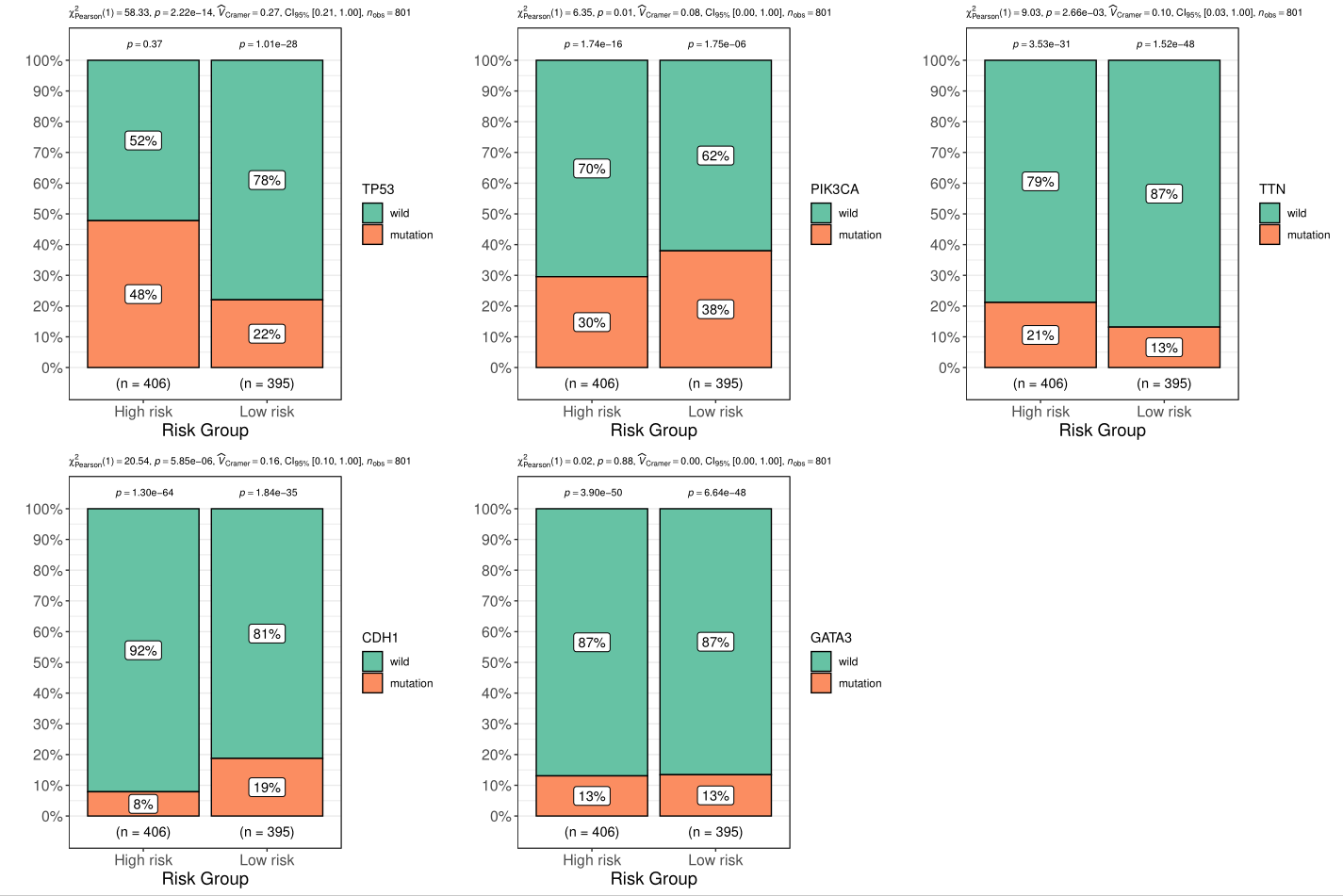


Fig. S6 Differences in the top 5 mutated genes between high and low risk groups.
